# Association of Private Equity Acquisition of Physician Practices With Changes in Health Care Spending and Utilization

**DOI:** 10.1001/jamahealthforum.2022.2886

**Published:** 2022-09-02

**Authors:** Yashaswini Singh, Zirui Song, Daniel Polsky, Joseph D. Bruch, Jane M. Zhu

**Affiliations:** 1 Department of Health Policy and Management, Bloomberg School of Public Health, Johns Hopkins University, Baltimore, Maryland; 2 Department of Health Care Policy, Harvard Medical School, Boston, Massachusetts; 3 Carey Business School, Johns Hopkins University, Baltimore, Maryland; 4 Division of General Internal Medicine, Oregon Health & Science University, Portland

## Abstract

**Importance:**

Private equity acquisitions of physician practices in the US have been increasing rapidly; however, the implications for health care delivery and spending are unclear.

**Objective:**

To examine changes in prices and utilization associated with private equity acquisitions of physician practices across multiple specialties.

**Design, Settings, and Participants:**

This was a difference-in-differences event study of US physician practices specialized in dermatology, gastroenterology, and ophthalmology that were acquired by private equity firms from 2016 to 2020. Within each specialty, each private equity−acquired (PE-acquired) practice was matched with as many as 5 control practices based on the preacquisition number of unique patients, encounters, risk score, share of services billed out-of-network, and spending. The PE-acquired practices were compared with matched controls through year 2 after acquisition, using a difference-in-differences event study. Data analyses were performed from March 2021 to February 2022.

**Exposures:**

Private equity acquisition of physician practices.

**Main Outcomes and Measures:**

Measures of spending and utilization, including the charge and price (amount paid) per claim, new and unique patients, and total encounters.

**Results:**

Compared with the 2874 control practices, the 578 PE-acquired physician practices exhibited an average increase of $71 (+20.2%) charged per claim (95% CI, 13.1%-27.3%; *P* < .001) and $23 (+11.0%) in the allowed amount per claim (95% CI, 5.6%-16.5%; *P* < .001). The PE-acquired practices increased their numbers of unique patients seen by 25.8% (95% CI, 15.8%-35.6%; *P* < .001) compared with control practices, driven by a 37.9% increase in visits by new patients (95% CI, 25.6%-50.2%; *P* < .001). In aggregate, their volume of encounters increased by 16.3% (95% CI, 1.0%-32.0%; *P* = .04) compared with the control group, with a 9.4% increase in the share of office visits for established patients that were billed as longer than 30 minutes (95% CI, 1.7%-17.0%; *P* = .02). No statistically significant changes in patient risk scores were found between PE-acquired practices and controls. Within specialties, we found modest differences along selected outcomes.

**Conclusions and Relevance:**

In this difference-in-differences study, private equity acquisition of physician practices in dermatology, gastroenterology, and ophthalmology were associated with differential increases in allowed amount and charges per claim, volume of encounters, and new patients seen, as well as some changes in billing and coding.

## Key Points

**Question:**

What are the implications of private equity acquisition of physician practices for health care spending and utilization?

**Findings:**

This difference-in-differences event study of 578 private equity−acquired dermatology, gastroenterology, and ophthalmology physician practices and 2874 similar independent practices found that spending, new and unique patient volume, and total encounters increased differentially compared with controls. The share of outpatient visits longer than 30 minutes increased, and there were modest differences along key outcomes within specialties.

**Meaning:**

The findings of this economic evaluation suggest that among a large commercially insured population, private equity acquisitions of physician practices were associated with increased health care spending and several measures of utilization.

## Teaser

This difference-in-differences study of private equity acquisition of physician practices examines changes in prices and utilization associated with acquisition.

## Introduction

Physician practices in the US are increasingly being acquired by institutional investors, notably private equity firms.^1^ This growth is facilitated by the physician practices’ growing need for capital, long-term practice uncertainty, and the promise of high purchase prices. Given the ongoing fragmentation in the physician practice market, a common strategy of private equity firms involves acquiring an established *platform* practice and adding on smaller practices to build market power, leverage economies of scale, capture referrals, and negotiate higher prices from payers.^2,3^ In general, private equity firms aim for annual returns exceeding 20% in a short investment period of 3 to 7 years.^2^ Although private equity acquisitions may bring technological and operational efficiencies into a practice, private equity’s short-term financial incentives and ownership models may have negative outcomes on health care access, quality, or spending.

Although the amount of rigorous evidence on how private equity acquisition affects physician practices is growing, it remains limited. Despite the rapid rise of private equity acquisition of nursing homes,^4,5,6,7^ hospital systems,^8,9,10,11^ and physician practices,^1,4^ little empirical evidence is available on how private equity influences utilization, spending, and other practice patterns among physicians. Private equity investment in nursing homes has been associated with an increase in short-term mortality and changes to staffing.^6,7^ Early research has found hospital acquisitions to be associated with a small increase in charge-to-cost ratio and net income,^10^ a higher likelihood of providing more profitable hospital-based services, and mixed effects on quality.^9,12^ It is unclear whether these findings generalize to physician practices, which have different business structures, service lines, and management practices.

Evidence of how private equity influences physician practices is limited to industry reports, academic opinion pieces,^2^ and largely descriptive studies within single specialties.^13,14,15,16,17,18^ A study that examined prices, utilization, and spending from 2013 to 2016 for dermatology services after private equity acquisitions found modest increases in prices and volume but not total spending.^19^ Important gaps in knowledge remain on how private equity affects pricing, service or patient composition, and billing practices within physician practices. Moreover, the extent to which acquisition-induced changes to practice patterns are specific to earlier acquisitions or to particular specialties is yet unknown.^20^

We address these gaps by examining changes in spending, utilization, and practice patterns following private equity acquisitions of physician practices from 2016 to 2020. Within a difference-in-differences event study design, we used novel data linkages of private equity acquisitions to commercial claims data to examine changes in practice patterns across 3 office-based specialties—dermatology, gastroenterology, and ophthalmology—with what is to our knowledge the largest number of acquisitions to date.^20^ Private equity has a common model for generating profit within this set of office-based, high-volume procedural specialties.^20^ This evaluation focuses on examining these potentially shared mechanisms of revenue generation.

## Methods

The study was approved by the institutional review board of Oregon Health & Science University. Informed consent was waived because we used only deidentified data from a large administrative claims database. The study followed the Consolidated Health Economic Evaluation Reporting Standards (CHEERS) reporting guideline.

The analytical sample involved multiple steps. First, we identified private equity acquisitions of physician practices; second, we identified multiple practice sites and physicians associated with each acquisition; and third, we linked this information with outcome measures constructed using claims data.

### Identifying Private Equity Acquisitions

We combined several data sources to identify physicians affiliated with private equity−acquired (PE-acquired) practices. First, to identify private equity acquisitions from 2016 to 2020 across dermatology, ophthalmology, and gastroenterology specialties, we used proprietary data from PitchBook Inc, a financial database that tracks mergers and acquisitions across industries and has been used by other studies to examine private equity in health care.^10,21^ We manually verified and expanded this list using a combination of press releases, industry reports, and physician practice websites. This manual verification process enabled us to identify stand-alone practice sites associated with each practice acquisition, as well as to account for changes in practice names following a private equity acquisition, using publicly available information.

### Identifying Physicians Affiliated With Practices

#### Data Sources

To identify individual physicians (medical doctors and doctors of osteopathic medicine) affiliated with both acquired and nonacquired practices, we used 2 IQVIA databases: the 2016 SK&A Office Based Physicians database and the 2019 OneKey database, which both use the same approach to identify and verify affiliations.^22^ Both data sets are independently verified with clinician-level information (eg, age, location, specialty, clinician credentials, National Provider Identifier [NPIs]) and practice-level information, including ownership and corporate affiliations, for 9.7 million health professionals in the US.^23,24^ A key advantage of these data sets is that they list distinct office locations belonging to each particular physician practice, allowing for precise identification of practice locations acquired by private equity firms.

#### Linking Acquisition Deals to Practices

We used probabilistic record linkage algorithms to link exact and non-exact records of practice names, addresses, and ownership information (eg, parent organization) in the 2019 OneKey data to reported acquisitions. For any unmatched deals, we manually matched a subset of acquisitions to the OneKey data by using public information online to verify practice locations and potential name changes for acquired practices. Because OneKey lists multiple office locations belonging to a particular practice, we included all practice sites associated with an acquisition. This linking strategy produced a match rate of 64% across all specialties and deal years, for a total of 2177 physicians in 601 PE-acquired practice locations.

To identify non−PE-acquired practices to serve as controls, we used OneKey to find a subset of independent physician practices in dermatology, gastroenterology, and ophthalmology, and excluded practices with other corporate ownership and hospital or health system affiliation.

#### Identifying Physicians Affiliated With Practices

To facilitate linkages to claims data, we identified physicians located at acquired and nonacquired practice sites. Because the linkages between physicians and their practice sites were available only in 2016 and 2019, we limited our analytic sample to physicians who remained at the same practice before and after acquisition in 2016 and 2019, respectively. Approximately 60% of physicians in private equity and non−private equity practices were at the same practice site in 2016 and 2019.

### Claims Data

#### Linking Physicians to Claims Data

Using NPIs, we linked our study sample of physicians to the FAIR Health National Private Insurance Claims (FAIR Health) patient deidentified claims data from January 1, 2015, through December 31, 2020. FAIR Health is an independent nonprofit that manages the largest database of commercial health insurance claims in the US. Prior studies have used FAIR Health claims data to examine imputed costs and utilization of care.^25,26^ Claim lines associated with each NPI were aggregated to the practice level, and the data were then further deidentified to mask any practice- or geographic-level identifiers, in compliance with FAIR Health data confidentiality policies. Therefore, NPI- or service-level analyses were not feasible. All analyses were conducted using FAIR Health’s deidentified data.

### Study Variables

Primary outcomes at the (physician) practice−(fiscal) quarter level were prices that reflected imputed allowed amounts and average charges obtained from the chargemaster. All primary and secondary outcome measures are defined in eTable 1 in the Supplement. To assess changes in utilization at the practice-quarter level, we measured (1) the number of unique patients; (2) the number of new patients; (3) total evaluation and management (E&M) visits; and (4) the total number of encounters (inclusive of all services, including procedures). Secondary outcomes included the median Hierarchical Category Conditions (HCC) risk score and the share of E&M visits for new and established patients with visits billed as longer than 30 minutes.

Finally, we separately examined total spending and volume of services for certain commonly billed services within specialties (eTable 2 in the Supplement). For dermatology, we focused on biopsy procedures, pathology services, and E&M visits; for gastroenterology, on procedures related to removal of polyps, tumors, and lesions, esophagogastroduodenoscopy, and E&M visits; and for ophthalmology, on cataract extractions, diagnostic imaging, and eye examinations.

### Statistical Analysis

We used a difference-in-differences design within an event study framework to compare outcomes in PE-acquired practices with those of matched controls. The PE-acquired practices were matched to controls in 2015, the year prior to any private equity acquisition in the study sample (see eTable 4 in the Supplement for matching methodology). Overall, 578 PE-acquired practices were matched to 2874 control practices, with 572 (98.5%) PE-acquired practices matched to a full set of 5 controls. We compared preacquisition practice characteristics for acquired practices with matched controls by examining parallel trends in preacquisition group differences and standardized mean differences to detect any remaining imbalance after matching.

In the event study analyses, event time 0 denoted the quarter of acquisition. We used data from 1 year before acquisition (event time –4, …, –1 quarter[s]) through 2 years after (event time +1, +2, …, +8 quarter[s]), with the quarter of acquisition as the reference period. The unit of analysis was the practice-quarter. A linear difference-in-differences regression model compared changes in outcomes of interest in PE-acquired practices with matched controls, before and after acquisition. We tested for differences in preacquisition trends between acquired practices and the control group by performing joint F tests of the hypothesis that preacquisition interactions between the treatment and time indicators were no different.

Adjusted differential change in percentage terms for outcomes of interest were derived by dividing estimates from a difference-in-differences model by the unadjusted preacquisition mean of the outcomes among PE-acquired practices. All regressions included specialty fixed-effects to account for time-invariant specialty-specific trends in the outcomes. To mitigate heteroskedasticity concerns driven by differences in practice size, all regressions were weighted by the average patient volume per practice over the study period. Standard errors were clustered at the level of the matched group (PE-acquired practice and its set of matched controls).

Several robustness checks assessed the sensitivity of results to: (1) matching only on specialty type to assess potential bias from matching on preintervention outcomes^27,28^; (2) unweighted regressions; (3) a longer preacquisition period of 8 quarters; (4) adding deidentified Metropolitan Statistical Area (MSA) fixed-effects; (5) adding deidentified practice and year fixed-effects to account for time-invariant differences across geographic areas and secular time trends; and (6) differential treatment timing and heterogeneous treatment effects.^29^ A falsification test estimated placebo treatment effects in the preacquisition period by changing the timing of private equity acquisition to a counterfactual quarter in the preacquisition period.^27,28^

Data analyses were conducted from March 2021 to February 2022 using Stata, release 16.1 (StataCorp LLC). Statistical tests were 2-tailed and *P* values < .05 were considered significant.

## Results

Table 1 reports summary statistics for 578 PE-acquired practices and their matched controls in 2015 (eTable 3 in the Supplement shows counts of acquisitions by specialty). Preacquisition differences between private equity and non−PE-acquired practices support the identifying assumption that group differences would remain constant in the absence of acquisition, as seen by statistically insignificant difference in levels (eTable 4 in the Supplement) as well as preacquisition parallel trends among primary outcomes (eFigure 1 in the Supplement).

**Table 1.** Characteristics of PE- and Non−PE-Acquired Physician Practices at Baseline, 2015

Characteristic	Mean (SD)		SMD
	PE-acquired	Non−PE-acquired^a^	
Physician practices, No.	578	2874	NA
Charge/claim, mean $	322 (258)	332 (326)	0.03
Allowed amount/claim, mean $	187 (136)	178 (136)	–0.06
Total No.			
Unique patients	94 (182)	88 (172)	–0.03
New patients	72 (136)	67 (132)	–0.03
Encounters	124 (237)	118 (224)	–0.02
E&M visits	75 (188)	72 (180)	–0.01
Share of E&M visits >30 min			
New patients	0.26 (0.15)	0.26 (0.21)	0.01
Established patients	0.19 (0.17)	0.18 (0.22)	–0.02
Patient HCC score, median	1.21 (1.05)	1.28 (1.10)	0.06

Abbreviations: E&M, evaluation and management; HCC, Hierarchical Condition Category; NA, not applicable; PE, private equity; SMD, standardized mean difference.

^a^ Independently owned physician practices identified using 5:1 caliper matching without replacement, requiring exact match on specialty and matches within 1 SD for continuous covariates (total No. of unique patients, total No. of encounters, median HCC score, and average allowed amount). The standardized bias was greatly reduced for all covariates after matching (eTable 4 in the Supplement) and was below 0.25 for all variables indicating negligible bias after inverse probability weighting.^37^

**Figure 1.**
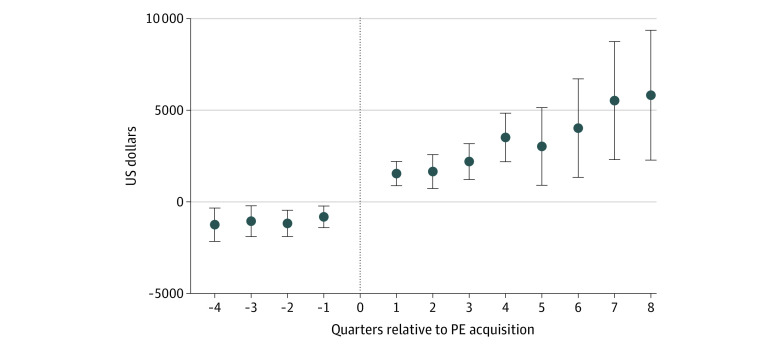
Changes in Total Spending per Practice Associated With Private Equity Acquisition, by Quarter Each point represents the coefficient obtained by estimating an event study regression. The vertical dashed line represents the quarter of acquisition (reference period). In the event study regression, we compared outcomes in PE-acquired practices with those matched controls up to 4 quarters before acquisition (event time −4, …, −1) and 8 quarters after (event time +1, +2, …, +8). The unit of analysis was the practice-quarter level, with event time 0 denoting the quarter of acquisition. Event study regressions included specialty fixed-effects, and standard errors are clustered at the level of the matched cohort. Increases in CIs in later quarters are explained by reductions in sample size.

In adjusted event study estimates, Figure 1 shows spending before and after private equity acquisition among acquired and control practices. Before acquisition, there were differences in spending at private equity and non-PE practices, although with trends appearing fairly parallel. After private equity acquisition, acquired practices demonstrated a consistent differential increase in spending through 8 quarters postacquisition.

**Figure 2.**
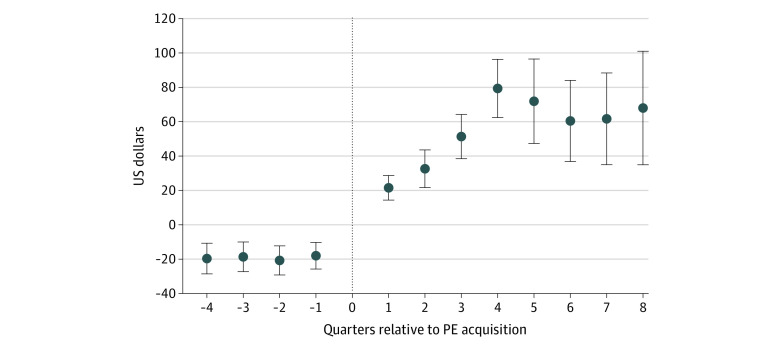
Changes in Allowed Amount per Claim Associated With Private Equity (PE) Acquisition of Physician Practice, by Quarter Each point represents the coefficient obtained by estimating an event study regression. The vertical dashed line represents the quarter of acquisition (reference period). In the event study regression, we compared outcomes in PE-acquired practices with those of matched controls up to 4 quarters before acquisition (event time −4, …, −1) and 8 quarters after (event time +1, +2, …, +8). The unit of analysis was the practice-quarter level, with event time 0 denoting the quarter of acquisition. Event study regressions include specialty fixed effects and are weighted by average patient volume per practice over the study period. Standard errors are clustered at the level of the matched cohort. Increases in CIs (error bars) in later quarters are explained by reductions in sample size.

Both average allowed amount per claim and average charges per claim increased for private equity practices in each of the 8 quarters after acquisition (Figure 2). Across all postacquisition quarters (Table 2), PE-acquired practices experienced a mean increase of $71 in charges per claim (95% CI, 13.1%-27.3%; *P* < .001) or a 20.2% increase, and an increase of $23 in allowed amount per claim (95% CI, 5.6%-16.5%; *P* < .001), which represented an 11.0% increase over the baseline.

**Table 2.** Differential Change in Practice Patterns for PE- and Non−PE-Acquired Physician Practices^a^

Variables	PE-acquired practices		Controls		Unadjusted	Adjusted		
	Before	After	Before	After	DiD	DiD	% (95% CI)	*P* value
Allowed amount/claim, $	206.0	285.0	201.0	260.0	20.0	22.8	11.0 (5.6 to 16.5)	<.001
**Utilization**								
Unique patient	105.0	147.0	93.0	108.0	27.0	27.1	25.8 (15.8 to 35.6)	<.001
New patient	57.0	89.0	47.0	57.0	22.0	21.6	37.9 (25.6 to 50.2)	<.001
**Practice patterns**								
Charge/claim, $	353.0	514.0	372.0	474.0	59.0	71.4	20.2 (13.1 to 27.3)	<.001
Patient HCC score	1.2	1.3	1.4	1.5	–0.1	0.01	1.0 (–2.4 to 4.8)	.64
E&M visit >30 min								
Established patient, %	0.2	0.2	0.2	0.2	0.0	0.01	9.4 (1.7 to 17.0)	.03
New patient, %	0.3	0.3	0.3	0.3	0.0	0.02	5.8 (–3.2 to 14.8)	.21
E&M visit	86.3	115.7	77.0	90.2	16.0	32.0	37.1 (–48.5 to 122.5)	.40
Encounters	138.1	191.1	123.8	143.5	33.0	91.0	16.3 (1.0 to 32.0)	.04

Abbreviations: DiD, difference in differences; E&M, evaluation and management; HCC, Hierarchical Condition Category; PE, private equity.

^a^ Unadjusted and adjusted differential changes in outcome variables averaged at the practice level for PE practices and matched controls. Adjusted regression coefficients were estimated using a linear DiD model that included specialty fixed-effects and was weighted by average patient volume per practice over the study period. Standard errors were clustered at the level of the matched cohort. Regressions with measures of patient volume as dependent variables (ie, total No. of unique patients and new patients) are unweighted. Adjusted percentage differential change was calculated by dividing the adjusted differential change obtained from the DiD regression, by the preacquisition mean for PE-acquired practices. DiD are between PE-acquired practices and controls or the differential change.

**Figure 3.**
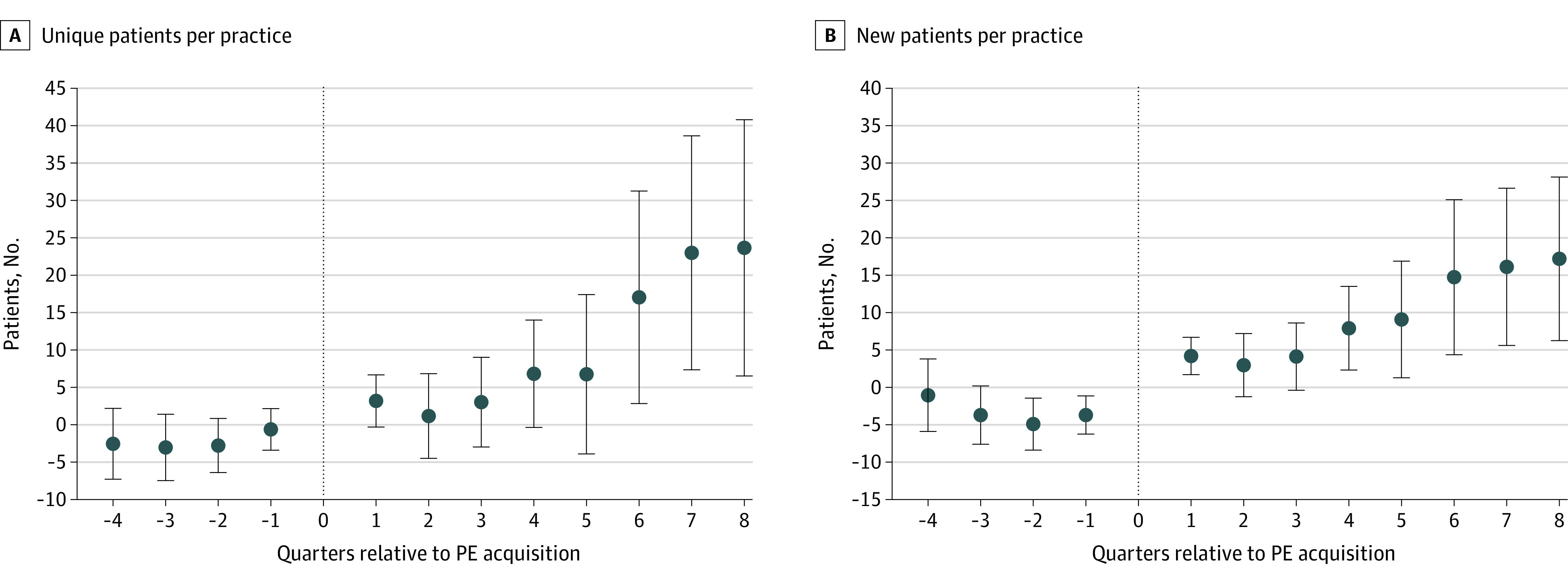
Changes in Utilization Associated With Private Equity (PE) Acquisition of Physician Practice, by Quarter Each point represents the coefficient obtained by estimating an event study regression. The vertical dashed line represents the quarter of acquisition (reference period). In the event study regression, we compared outcomes in PE-acquired practices with those of matched controls up to 4 quarters before acquisition (event time −4, …, −1) and 8 quarters after (event time +1, +2, …, +8). The unit of analysis was the practice-quarter level, with event time 0 denoting the quarter of acquisition. Event study regressions include specialty fixed-effects. Standard errors are clustered at the level of the matched cohort. Increases in CIs (error bars) in later quarters are explained by reductions in sample size.

The increases in allowed amount and charges per claim after private equity acquisition were accompanied by increases in patient utilization in each of the 8 quarters after acquisition (Figure 3). Averaged across all postacquisition quarters (Table 2), the mean number of unique patients increased by 25.8% (95% CI, 15.8%-35.6%; *P* < .001), primarily driven by increased visits for new patients, which rose by 37.9% (95% CI, 25.6%-50.2%; *P* < .001). The total number of encounters increased by 16.3% (95% CI, 1.0%-32.0%; *P* = .04) after private equity acquisition. The number of E&M visits increased by 37.1% but was not statistically significant.

Table 2 shows the preacquisition and postacquisition means, along with differential changes, in our outcomes for PE-acquired practices and matched controls. In addition to the outcomes mentioned, PE-acquired practices experienced a 5.4% reduction in share of total spending on out-of-network services (95% CI, –9.5% to –1.0%; *P* = .01) and a 9.4% increase in the share of E&M visits for established patients that were billed as longer than 30 minutes (95% CI, 1.7% to 17.0%; *P* = .02) compared with the controls. As the results in Table 2 show, a 5.8% increase in the share of E&M visits for new patients billed as longer than 30 minutes was not statistically significant (*P* = .21); neither was the differential change in median patient HCC scores (*P* = .64). These trends persisted through 8 quarters after acquisition (eFigure 2 in the Supplement).

Joint F tests to assess differences in preacquisition trends showed differences in outcomes of interest between PE-acquired and non−PE-acquired practices (eTable 5 in the Supplement). These findings were robust to alternative matching approaches using exact matching on specialty type only (eTables 6 and 7 in the Supplement), unweighted regression specifications (eTable 8 in the Supplement), longer preacquisition period (eTable 9 in the Supplement), adding MSA fixed-effects (eFigure 3 in the Supplement), adding practice and year fixed-effects (eFigure 4 in the Supplement), as well as using the Callaway and Sant’Anna estimator^29^ for differential treatment timing and heterogeneous treatment effects (eFigure 5 in the Supplement) and falsification tests (eFigure 6 in the Supplement).

Specialty-specific analyses demonstrated some modest variation along select outcomes of interest. For example, although all specialties saw an increase in unique and new patients and charges, allowed amounts per claim increased for ophthalmology and gastroenterology after private equity acquisition, but not for dermatology. Additionally, before acquisition, gastroenterology practices had a higher share of patients with higher-intensity E&M visits as compared with dermatology and ophthalmology practices. Postacquisition, the share of established patients with higher-intensity visits increased for dermatology and gastroenterology, but not for ophthalmology (eTables 10-12 and eFigures 7-9 in the Supplement). Lastly, procedure-specific event study plots demonstrated increases in volumes and spending across select services, with variation across specialties (eg, in dermatology, increases in E&M and pathology service volume and total spending, but not biopsy procedures; in gastroenterology, increases in E&M and esophagogastroduodenoscopy volume and total spending, but not polyp or tumor removal; in ophthalmology, diagnostic imaging volume and total spending, but not cataract removals or eye examinations).

## Discussion

Across 578 physician practices in dermatology, gastroenterology, and ophthalmology, private equity acquisition was associated with increases in health care spending and several measures of utilization, and some evidence of greater intensity of care. Importantly, these data did not allow us to distinguish between the reasons for higher spending associated with private equity acquisition. For example, increases in allowed amount per claim could be explained by service mix changing toward higher-priced services; changes in billing practices, including more efficient charge capture or intensive coding; or higher negotiated prices for each service. Despite being unable to assess changes in service mix, we did detect an increase in coding intensity of E&M visits for both new and established patients. In sensitivity analyses using commonly billed procedure codes within each specialty, we also observed increases in volumes of certain procedural and ancillary services as 1 potential mechanism for these findings. Other researchers have found higher prices after private equity acquisition.^10,19^ Taken together, these results provide further empirical support for the business strategies that private equity firms may pursue in physician practice markets.

Private equity acquisition was also associated with increased patient utilization, both by bringing established patients back more often as well as by increasing the numbers of new patients at a practice. Increases in the number of visits were consistent with a long-standing strategy to maximize revenue within a largely fee-for-service delivery system. Given that our study design held constant the physicians at each practice before and after acquisition, increased patient utilization per practice was unlikely to be the result of new physician hires. Instead, these findings may reflect changes in management and practice operations, such as expanding practice hours, branding and advertising, or broadening referral networks.^2,20,30^ Alternately, increased patient volume may also reflect overutilization of profitable services and/or unnecessary or low-value care, which could raise health care spending without commensurate patient benefits.^2,20,30^

Despite finding an increase in coding intensity of E&M visits for both new and established patients, we were unable to attribute these findings to more specific mechanisms. Increased coding intensity may reflect managerial changes to coding and billing practices associated with more efficient charge capture^31^ facilitated by electronic health record use or other technology adoption, and correction of missing, erroneous, and/or lagged charges.^32^ On the contrary, coding intensity could also reflect upcoding to optimize revenues, which can produce overpayments for services rendered.^31^ Given that we did not find evidence of selective sorting of patients with low-complexity needs after private equity acquisition, it is unlikely that differences in coding intensity for E&M visits reflect true differences in patient health. Whether differences in coding intensity represent practice efficiency gains vs upcoding postacquisition warrants further study.

This study focused on common and general mechanisms of revenue generation across office-based procedural specialties that, to our knowledge, have not been examined in the literature to date. However, private equity acquisitions may induce specialty-specific changes, including increases in the intensity of specific procedures, such as skin biopsy procedures in dermatology^19^ or colonoscopy examinations in gastroenterology. Other researchers have also identified separate mechanisms that are used by private equity firms to target hospital-based physicians vs primary care practices operating under value-based payment arrangements.^20^ Our findings suggest there are common postacquisition changes across specialties, as well as some modest variation in outcomes of interest across selected commonly billed procedures within each specialty. Some variation could be explained by underlying differences in billing practices, patient populations, and service mix offered across specialties, which private equity may exploit in slightly different ways. Nonetheless, understanding the mechanisms of private equity that increase profits—both general approaches across specialties, and specific approaches within specialties—is critically important for thoughtful policy responses to potentially adverse effects.

### Limitations

This study had several limitations. First, some private equity acquisitions may have been missed, although our acquisition counts were consistent with those of other single-specialty studies.^13,14,19^ Use of OneKey’s secondary data may also include sampling and/or measurement error.^22^ Second, acquired practices may differ from independent practices in unobserved ways, as demonstrated by preintervention differences. Third, we were unable to include ancillary (eg, optometrists) and advanced practitioners (eg, nurse practitioners) because they often bill under supervisory physicians, it is possible that more advanced practitioner staffing may have contributed to the observed increases in patient volume and utilization.^33^ Fifth, deidentification and aggregation of FAIR Health data precluded NPI- or service-level analyses. Sixth, given the recency of these private equity acquisitions, our follow-up period included only 8 quarters postacquisition, and some of our specialty-specific estimates may be limited in statistical power owing to smaller numbers of acquisitions. Finally, given our focus on a commercially insured population, the findings of this study may not be generalizable to other payers.

## Conclusions

In this difference-in-differences study, private equity acquisitions of physician practices were associated with increases in health care spending and utilization, along with some changes to practice patterns. Private equity ownership of physician practices has added a distinctly private and market-driven influence to the broader trends in corporate consolidation of physicians by health systems and insurers.^2,3,20,34,35^ This study contributes evidence for potential overutilization and higher spending on care that will be important for policy makers to monitor.^20^ Other consequences of private equity acquisitions, including quality of care, patient satisfaction,^36^ and the physician practice experience, remain key areas for future research.

## Notes

Supplement. eTable 1. Definitions of outcomes eTable 2. List of CPTs eTable 3. Counts of practices and physicians identified as PE-acquired, by specialty and year eTable 4. Characteristics of PE acquired and non-PE practices, before and after matching, 2015 eFigure 1. Unadjusted trends in primary outcomes for PE practices and matched controls eFigure 2. Changes in practice patterns associated with PE acquisition, by quarter eTable 5. Joint F-test of Pre-acquisition Trends eTable 6. Characteristics of PE acquired and non-PE practices, 2015 (Matching on specialty type only) eTable 7. Differential change in practice patterns for PE practices and matched controls (Matching on specialty type only) eTable 8. Differential change in practice patterns for PE practices and matched controls (Unweighted) eTable 9. Differential change in practice patterns for PE practices and matched controls (8 quarter preacquisition period) eFigure 3. Changes in practice patterns associated with PE acquisition, by quarter (MSA fixed effects) eFigure 4. Changes in practice patterns associated with PE acquisition, by quarter (practice and year fixed effects) eFigure 5. Changes in practice patterns associated with PE acquisition, by quarter (Callaway & Sant’Anna (2021)) eFigure 6. Changes in practice patterns associated with PE acquisition, by quarter (Falsification test) eTable 10. Differential change in practice patterns for PE practices and matched controls (Dermatology only) eTable 11. Differential change in practice patterns for PE practices and matched controls (Gastroenterology only) eTable 12. Differential change in practice patterns for PE practices and matched controls (Ophthalmology only) eFigure 7. Differential change in volume and spending of select services – Dermatology eFigure 8. Differential change in volume and spending of select services – Gastroenterology eFigure 9. Differential change in volume and spending of select services – Ophthalmology
