# Supplementary material for: Association of Private Equity Acquisition of Physician Practices With Changes in Health Care Spending and Utilization
Source: JAMA Health Forum. 2022 Sep 2;3(9):e222886. doi: 10.1001/jamahealthforum.2022.2886 (PMC9440392; doi:10.1001/jamahealthforum.2022.2886)
Supplement: Supplement. — eTable 1. Definitions of outcomes eTable 2. List of CPTs eTable 3. Counts of practices and physicians identified as PE-acquired, by specialty and year eTable 4. Characteristics of PE acquired and non-PE practices, before and after matching, 2015 eFigure 1. Unadjusted trends in primary outcomes for PE practices and matched controls eFigure 2. Changes in practice patterns associated with PE acquisition, by quarter eTable 5. Joint F-test of Pre-acquisition Trends eTable 6. Characteristics of PE acquired and non-PE practices, 2015 (Matching on specialty type only) eTable 7. Differential change in practice patterns for PE practices and matched controls (Matching on specialty type only) eTable 8. Differential change in practice patterns for PE practices and matched controls (Unweighted) eTable 9. Differential change in practice patterns for PE practices and matched controls (8 quarter preacquisition period) eFigure 3. Changes in practice patterns associated with PE acquisition, by quarter (MSA fixed effects) eFigure 4. Changes in practice patterns associated with PE acquisition, by quarter (practice and year fixed effects) eFigure 5. Changes in practice patterns associated with PE acquisition, by quarter (Callaway & Sant’Anna (2021)) eFigure 6. Changes in practice patterns associated with PE acquisition, by quarter (Falsification test) eTable 10. Differential change in practice patterns for PE practices and matched controls (Dermatology only) eTable 11. Differential change in practice patterns for PE practices and matched controls (Gastroenterology only) eTable 12. Differential change in practice patterns for PE practices and matched controls (Ophthalmology only) eFigure 7. Differential change in volume and spending of select services – Dermatology eFigure 8. Differential change in volume and spending of select services – Gastroenterology eFigure 9. Differential change in volume and spending of select services – Ophthalmology [file jamahealthforum-e222886-s001.pdf]

Supplemental Online Content

Singh Y, Song Z, Polsky D, Bruch JD, Zhu JM. Association of private equity acquisition of physician practices with changes in health care spending and utilization. *JAMA Health Forum.* 2022;3(9):e222886. doi:10.1001/jamahealthforum.2022.2886

eTable 1. Definitions of outcomes

eTable 2. List of CPTs

eTable 3. Counts of practices and physicians identified as PE-acquired, by specialty and year

eTable 4. Characteristics of PE acquired and non-PE practices, before and after matching, 2015

eFigure 1. Unadjusted trends in primary outcomes for PE practices and matched controls

eFigure 2. Changes in practice patterns associated with PE acquisition, by quarter

eTable 5. Joint F-test of Pre-acquisition Trends

eTable 6. Characteristics of PE acquired and non-PE practices, 2015 (Matching on specialty type only)

eTable 7. Differential change in practice patterns for PE practices and matched controls (Matching on specialty type only)

eTable 8. Differential change in practice patterns for PE practices and matched controls (Unweighted)

eTable 9. Differential change in practice patterns for PE practices and matched controls (8 quarter preacquisition period)

eFigure 3. Changes in practice patterns associated with PE acquisition, by quarter (MSA fixed effects)

eFigure 4. Changes in practice patterns associated with PE acquisition, by quarter (practice and year fixed effects)

eFigure 5. Changes in practice patterns associated with PE acquisition, by quarter (Callaway & Sant'Anna (2021))

eFigure 6. Changes in practice patterns associated with PE acquisition, by quarter (Falsification test)

© 2022 Singh Y et al. *JAMA Health Forum.*

eTable 10. Differential change in practice patterns for PE practices and matched controls (Dermatology only)

eTable 11. Differential change in practice patterns for PE practices and matched controls (Gastroenterology only)

eTable 12. Differential change in practice patterns for PE practices and matched controls (Ophthalmology only)

eFigure 7. Differential change in volume and spending of select services – Dermatology

eFigure 8. Differential change in volume and spending of select services – Gastroenterology

eFigure 9. Differential change in volume and spending of select services – Ophthalmology

This supplemental material has been provided by the authors to give readers additional information about their work.

© 2022 Singh et al. *JAMA Health Forum.*

eTable 1. Definitions of outcomes

Variable	Definition
Charge per claim	Sum of all chargemaster prices for a practice divided by the number of claims per practice, in a given quarter.
Allowed amount per claim	Sum of all imputed allowed amounts for a practice divided by the number of claims per practice, in a given quarter.
Total no. of unique patients	Number of unique patients, in a given practice-quarter.
Total no. of new patients	Number of patients in a practice who were not seen in the two years prior to the date of service, in a given practice-quarter.
Total no. of encounters	Total number of encounters, including claims with multiple encounters without any restrictions on type of service or care received (e.g., procedures, ancillary services, etc.)
Total no. of E&M visits	Total number of claims for Evaluation and Management (E&M) visits (Current Procedural Terminology (CPT) codes 99201-99215, 99241-99255, and 99499) in a given practice quarter.
Share of E&M visits >30 min (new patients)	Total number of E&M visits for established patients (CPT codes 99201-99205) billed as longer than 30 minutes (CPT codes 99204-99205).
Share of E&M visits >30 min (est. patients)	Total number of E&M visits for established patients (CPT codes 99211-99215) billed as longer than 30 minutes (CPT codes 99214-99215).
Median Patient HCC Score	Median HCC score for all patients in a practice-year, using the HHS-HCC algorithm that uses claims from the year prior and the current year to estimate risk scores.

Notes: All outcomes are calculated by FAIR Health and aggregated to the practice-quarter level in compliance with FAIR Health confidentiality policies. Outcomes are averaged across claims among physicians who remained at each practice before and after acquisition.

© 2022 Singh et al. *JAMA Health Forum.*

eTable 2. List of CPTs

Service Category	CPTs
Dermatology - Biopsies	11100 – 11107<br>11300 – 11313<br>40490, 69100
Dermatology - Pathology	88304 – 88305<br>88312, 88314
Dermatology – E&M	99201 – 99215
Gastroenterology - Removal of Tumors, Polyps and Lesions	43216, 43250, 44365, 44392, 45333, 45384, 43217, 43251, 44364, 44394, 45338, 45385, 43229, 43270, 44369, 44401, 45346, 45388
Gastroenterology - EGD	43200 – 43205<br>43211 – 43217<br>43226 – 43227<br>43235 – 43236<br>43220, 43229, 43233, 43239, 43241<br>43243 – 43251<br>43254 – 43255<br>43266, 43270<br>91110 – 91111
Gastroenterology – E&M	99201 – 99215
Ophthalmology – Cataract Extraction	66982 – 66984
Ophthalmology – Diagnostic Imaging	92250<br>92132 – 92134
Ophthalmology – Eye Exams	92002 – 99204<br>92012 - 92014

© 2022 Singh et al. *JAMA Health Forum.*

eTable 3. Counts of practices and physicians identified as PE-acquired, by specialty and year

	**Count of Practices**				**Count of Physicians**			
	**Derm**	**Gastro**	**Ophtho**	**Total**	**Derm**	**Gastro**	**Ophtho**	**Total**
2016	33	23	1	57	64	87	2	**153**
2017	80	12	25	117	149	34	51	**234**
2018	72	46	62	180	122	119	139	**380**
2019	48	78	97	223	92	287	170	**549**
2020	0	1	0	1	0	171	0	**171**
**All years**	**233**	**160**	**185**	**578**	**427**	**698**	**362**	**1487**

Notes: Counts of practices represent the number of practices identified as being acquired by PE in the study period. Counts of physicians represent the number of unique National Provider Identifiers (NPIs) for MDs or DOs identified as being acquired by PE in the study period. Derm=Dermatology, Gastro=Gastroenterology, Ophtho=Ophthalmology.

© 2022 Singh et al. *JAMA Health Forum.*

eTable 4: Characteristics of PE acquired and non-PE practices, before and after matching, 2015

	Before Matching			After Matching		
	Mean (SD)		Standardized Mean Difference	Mean (SD)		Standardized Mean Difference
	PE (N=578)	Non-PE (N=2,874)		PE (N=578)	Non-PE (N=2,874)	
**Charge per claim**	323 (258)	434 (936)	0.16	322 (258)	332 (326)	0.03
**Allowed amount per claim**	187(135)	216 (340)	0.11	187 (136)	178 (136)	-0.06
**Total no. of unique patients**	99 (197)	80 (164)	-0.10	94 (182)	88 (172)	-0.03
**Total no. of new patients**	76 (151)	60 (124)	-0.10	72 (136)	67 (132)	-0.03
**Total no. of encounters**	131 (261)	107 (215)	-0.10	124 (237)	118 (224)	-0.02
**Total no. of E&M visits**	80 (202)	63 (161)	-0.08	75 (188)	72 (180)	-0.01
**Share of E&M visits >30 min (new patients)**	0.26 (0.15)	0.26 (0.22)	0.02	0.26 (0.15)	0.26 (0.21)	0.01
**Share of E&M visits >30 min (est. patients)**	0.18 (0.17)	0.16 (0.20)	-0.13	0.19 (0.17)	0.18 (0.22)	-0.02
**Median Patient HCC Score**	1.26 (1.45)	1.38 (1.78)	0.07	1.21 (1.05)	1.28 (1.10)	0.06

Notes: Baseline characteristics for private equity acquired and non-acquired practices in 2015. Non-acquired practices represent independently owned physician practices identified using 5:1 caliper matching without replacement. Matching algorithm requires exact match on specialty (dermatology, ophthalmology, gastroenterology), and matches within 1 standard deviation for continuous covariates (total number of unique patients, total number of encounters, median HCC score, and average allowed amount). E&M=Evaluation and Management, HCC=Hierarchical Condition Category.

© 2022 Singh et al. *JAMA Health Forum.*

eFigure 1: Unadjusted trends in primary outcomes for PE practices and matched controls

(a) Total spending per practice

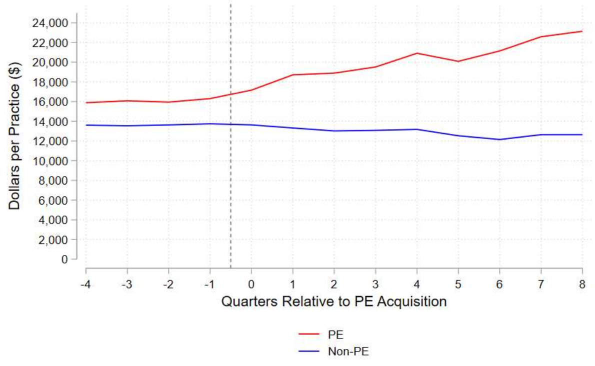

(b) Allowed amount per claim

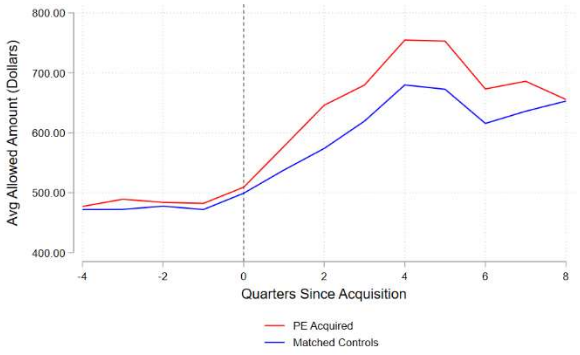

(c) Charges per claim

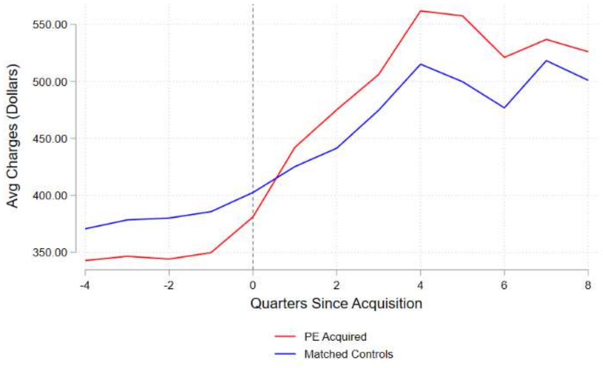

(d) Total number of E&M visits

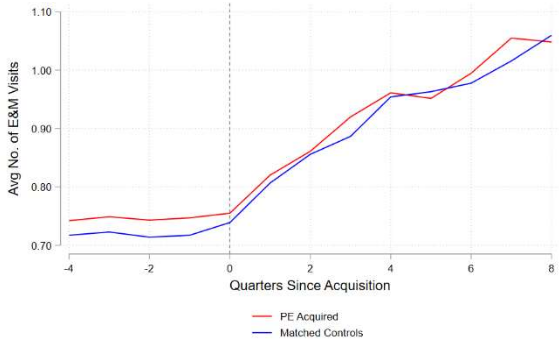

(e) Total number of encounters

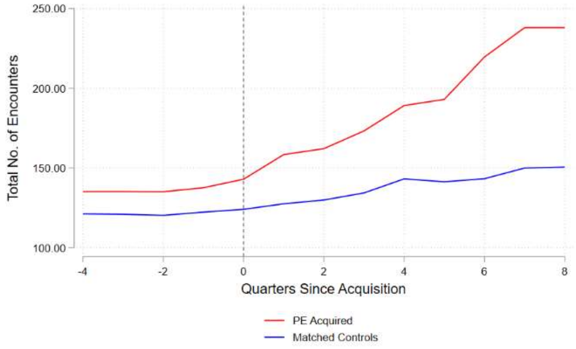

(f) Median HCC score

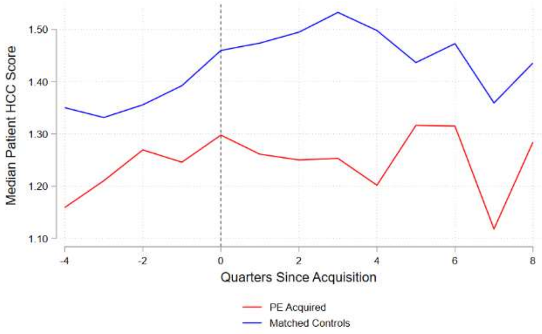

(g) E&M visits longer than 30 min (New)

(h) E&M visits longer than 30 min (Est)

© 2022 Singh et al. *JAMA Health Forum.*

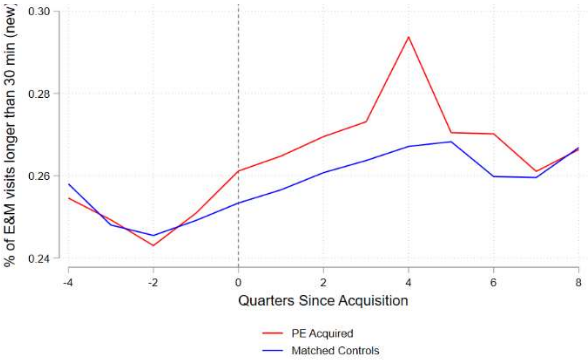

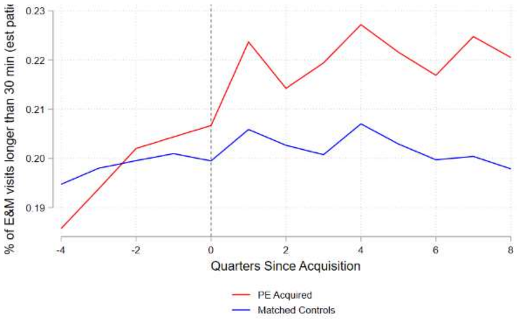

Notes: eFigure 1 presents unadjusted outcomes of interest for PE practices and matched controls. For matched controls, the quarter of acquisition represents the quarter in which the PE practice in the match cohort was acquired. The vertical dash line represents the quarter of acquisition.

© 2022 Singh et al. *JAMA Health Forum.*

eFigure 2 (a)-(f): Changes in practice patterns associated with PE acquisition, by quarter

(a) Charges per claim

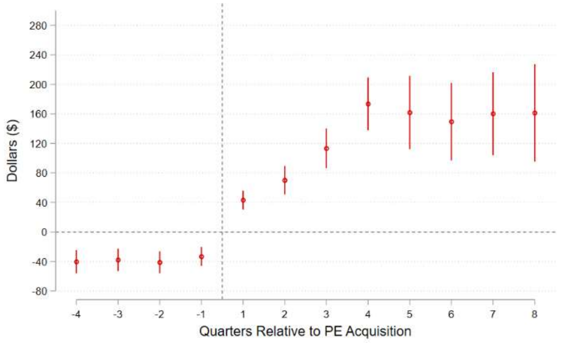

(b) Total number of encounters

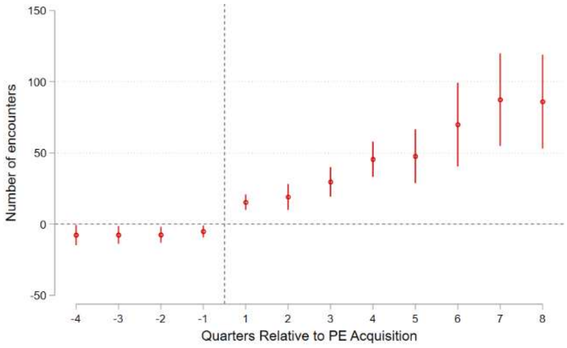

(c) Total number of E&M visits

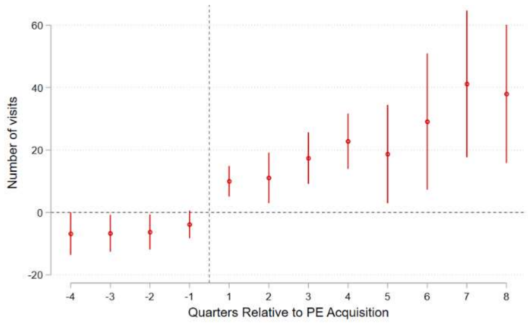

(d) Median HCC score

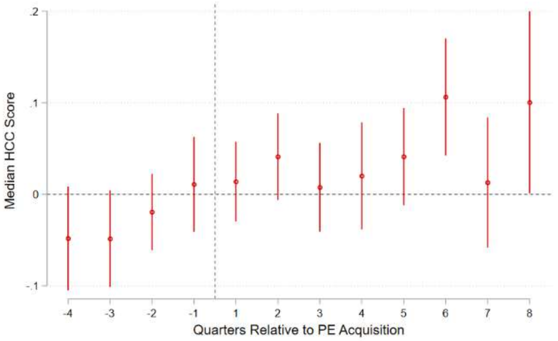

(e) E&M visits longer than 30 min (New)

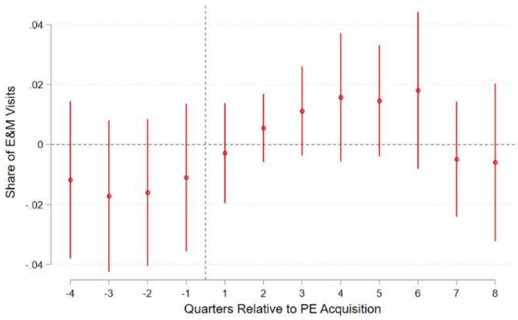

(f) E&M visits longer than 30 min (Established)

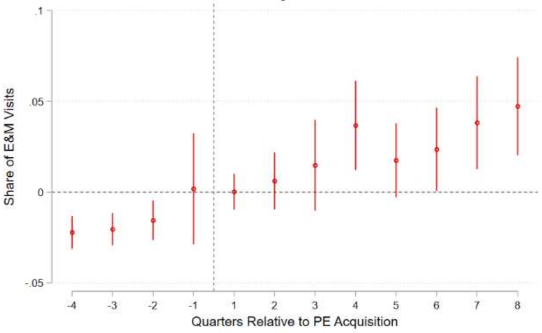

Notes: eFigure 2 presents event study coefficients corresponding to PE acquisition of physician practices. In the event study regression, we compared outcomes in PE-acquired practices with that of matched controls. Each point in the respective figure panels represents the coefficient obtained by estimating an event study regression which includes specialty fixed effects and with the unit of analysis at the practice-quarter level. The vertical dash line represents the quarter of acquisition that serves as the reference period. Event time 0 denoted the quarter of acquisition. We looked back up to 4 quarters prior to acquisition (event time -4, …, -1) and 8 quarters after (event time +1, +2, …, +8). Standard errors are clustered at the level of the matched cohort. Increases in confidence intervals in later quarters are explained by reductions in sample size.

© 2022 Singh et al. *JAMA Health Forum.*

eTable 5. Joint F-test of Pre-acquisition Time Period, 2012-2017

Outcome	F-statistic (P-value)
Total spending	0.89 (0.44)
Charge per claim	1.84 (0.13)
Allowed amount per claim	0.97 (0.40)
Total no. of unique patients	0.90 (.044)
Total no. of new patients	3.46 (0.01)
Total no. of encounters	0.51 (0.67)
Total no. of E&M visits	0.81 (0.49)
Share of E&M visits >30 min (new patients)	0.93 (0.42)
Share of E&M visits >30 min (est. patients)	1.05 (0.37)
Median Patient HCC Score	1.98 (0.11)

Note: Authors' analysis of FAIR Health data. We tested for differences in pre-acquisition trends between PE-acquired and non-PE practices by performing a joint F-Test of the hypothesis that pre-acquisition interactions between the treatment and time indicators were no different.

© 2022 Singh et al. *JAMA Health Forum.*

eTable 6: Characteristics of PE acquired and non-PE practices in 2015 (Matching on specialty type only)

	Mean (SD)		Standardized Mean Difference
	PE Practices<br><br>(N=579)	Non-PE Practices<br>(N=2,890)	
Average charges per claim	322 (258)	433 (772)	0.19
Average allowed amount per claim	187 (136)	219 (288)	0.14
Total no. of unique patients	96 (188)	88 (179)	-0.04
Total no. of new patients	73 (141)	67 (137)	-0.04
Total no. of encounters	126 (249)	118 (236)	-0.03
Total no. of E&M visits	78 (200)	71 (185)	-0.03
Share of E&M visits >30 min (New patients)	0.26 (0.15)	0.26 (0.21)	0.001
Share of E&M visits >30 min (Established patients)	0.19 (0.17)	0.18 (0.22)	-0.01
Median Patient HCC Score	1.25 (1.45)	1.54 (2.01)	0.16

Notes: Baseline characteristics for PE acquired and non-acquired practices in 2015, prior to any acquisition in our analytic sample, based on exact matching within specialty type only. Of the 578 PE-acquired facilities in our sample, 576 (99.1%) were matched with a full set of five matched controls. E&M=Evaluation and Management, HCC=Hierarchical Condition Category.

© 2022 Singh et al. *JAMA Health Forum.*

eTable 7: Differential change in practice patterns for PE and control practices (Matching on specialty type only)

	PE		Controls		Unadjusted	Adjusted		
	Pre	Post	Pre	Post	Diff-in-diff	Diff-in-diff	% [95% C.I.]	p-value
Allowed amount per claim, $	206	285	227	294	12.0	17.8	8.6 [2.7, 14.6]	< 0.01
**Utilization**								
Unique patients	104	147	91	105	29.0	28.4	28.4 [17.3, 37.3]	< 0.01
New patients	56	88	46	56	22.0	22.6	22.6 [27.9, 53.0]	< 0.01
**Practice Patterns**								
Charges per claim, $	353	512	436	576	19.0	58.8	16.7 [8.7, 24.7]	< 0.01
Patient HCC score	1.29	1.30	1.52	1.55	-0.02	0.02	1.6 [-1.8, 6.1]	0.29
E&M visits >30 minutes (established patients), %	0.20	0.22	0.20	0.20	0.02	0.02	10.0 [3.9, 18.7]	< 0.01
E&M visits >30 minutes (new patients), %	0.25	0.27	0.26	0.27	0.01	0.01	4.0 [-2.8, 12.9]	0.21
Evaluation & Management visits	85.8	115.4	74.7	86.9	17.5	30.2	35.2 [-52.4, 122.8]	0.43
Encounters	137.0	190.5	122.1	140.0	35.8	89.6	65.4 [0.1, 130.7]	0.05

Notes: eTable 6 shows unadjusted and adjusted differential changes in outcome variables averaged at the practice level for PE practices and matched controls, based on exact matching on specialty type alone. Adjusted regression coefficients are estimated using a linear difference-in-differences model that includes specialty fixed effects and is weighted by average patient volume per practice over the study period. Standard errors are clustered at the level of the matched cohort. Regressions with measures of patient volume as dependent variables (i.e., total number of unique patients and total number of new patients) are unweighted. Adjusted percentage differential change is calculated by dividing the adjusted differential change, obtained from the difference-in-differences regression, by the preacquisition mean for PE-acquired practices. E&M=Evaluation and Management, HCC=Hierarchical Condition Category. Diff-in-diff is the difference in differences between PE and controls or the differential change.

© 2022 Singh et al. *JAMA Health Forum.*

eTable 8: Differential change in practice patterns for PE and control practices (Unweighted)

	PE		Controls		Unadjusted	Adjusted		
	**Pre**	**Post**	**Pre**	**Post**	**Diff-in-diff**	**Diff-in-diff**	**%<br>[95% C.I.]**	**p-value**
Allowed amount per claim, $	206.0	285.0	201.0	260.0	20.0	19.7	9.6<br>[5.2, 14.0]	<0.01
**Utilization**								
Unique patients	105.0	147.0	93.0	108.0	27.0	27.1	25.8<br>[15.8, 35.6]	<0.01
New patients	57.0	89.0	47.0	57.0	22.0	21.6	37.9<br>[25.6, 50.2]	<0.01
**Practice Patterns**								
Charges per claim, $	353.0	514.0	372.0	474.0	59.0	57.1	16.2<br>[10.6, 21.8]	<0.01
Patient HCC score	1.2	1.3	1.4	1.5	-0.1	-0.08	-6.6<br>[-13.2, -0.1]	0.04
E&M visits >30 minutes (established patients), %	0.2	0.2	0.2	0.2	0.0	0.02	9.6<br>[4.1, 15.0]	<0.01
E&M visits >30 minutes (new patients), %	0.3	0.3	0.3	0.3	0.0	0.01	2.7<br>[-1.1, 6.5]	0.16
Evaluation & Management visits	86.3	115.7	77.0	90.2	16	16.3	18.8<br>[7.1, 30.6]	<0.01
Encounters	138.1	191.1	123.8	143.5	33	33.4	24.2<br>[14.5, 33.8]	<0.01

Notes: Unadjusted and adjusted differential changes in outcome variables averaged at the practice level for PE practices and matched controls, using unweighted linear difference-in-differences model that includes specialty fixed effects. Standard errors are clustered at the level of the matched cohort. Adjusted percentage differential change is calculated by dividing the adjusted differential change obtained from the difference-in-differences regression, by the preacquisition mean for PE-acquired practices. E&M=Evaluation and Management, HCC=Hierarchical Condition Category. Diff-in-diff is the difference in differences between PE and controls or the differential change.

© 2022 Singh et al. *JAMA Health Forum.*

eTable 9: Differential change in practice patterns for PE and control practices (8 quarter preacquisition period)

	PE		Controls		Unadjusted	Adjusted		
	Pre	Post	Pre	Post	Diff-in-diff	Diff-in-diff	% [95% C.I.]	p-value
Allowed amount per claim, $	199	285	197	260	23	25.0	12.6 [6.8, 18.3]	< 0.01
**Utilization**								
Unique patients	102	147	91	108	28	28.2	27.7 [16.6, 38.7]	< 0.01
New patients	57	89	47	57	22	22.3	39.2 [25.8, 52.6]	< 0.01
**Practice Patterns**								
Charges per claim, $	341.0	514.0	365.0	474.0	64.0	76.0	22.3 [14.8, 29.8]	< 0.01
Patient HCC score	1.3	1.3	1.4	1.5	-0.1	0.00	0.4 [-3.8, 4.6]	0.85
E&M visits >30 minutes (established patients), %	0.20	0.22	0.20	0.20	0.02	0.02	11.1 [2.8, 19.4]	< 0.01
E&M visits >30 minutes (new patients), %	0.25	0.27	0.26	0.27	0.01	0.02	6.3 [-3.7, -16.2]	0.21
Evaluation & Management visits	81.7	115.7	74.7	90.2	18.5	35.5	43.5 [-60.1, 147.1]	0.41
Encounters	134.0	191.1	121.0	143.5	34.7	87.9	65.6 [-9.0, 140.1]	0.08

Notes: eTable 8 shows unadjusted and adjusted differential changes in outcome variables averaged at the practice level for PE practices and matched controls, based on a sensitivity analysis that extended the pre-acquisition period to 8 quarters, compared to 4 quarters in the main analysis. Post-acquisition period remains 8 quarters. E&M=Evaluation and Management, HCC=Hierarchical Condition Category. Diff-in-diff is the difference in differences between PE and controls or the differential change.

© 2022 Singh et al. *JAMA Health Forum.*

eFigure 3: Changes in practice patterns associated with PE acquisition, by quarter (MSA fixed effects)

(a) Total spending per practice

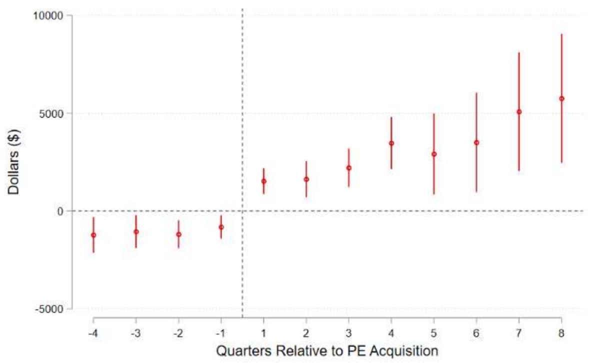

(b) Allowed amount per claim

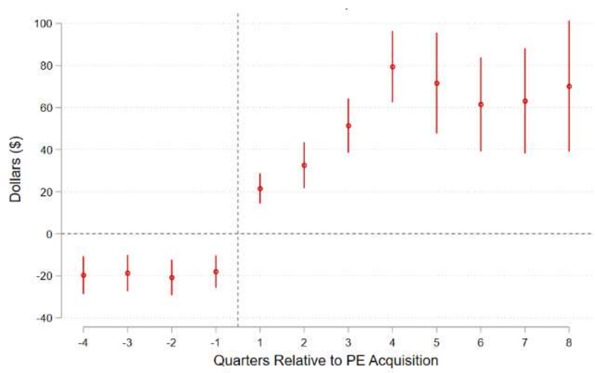

(c) Charges per claim

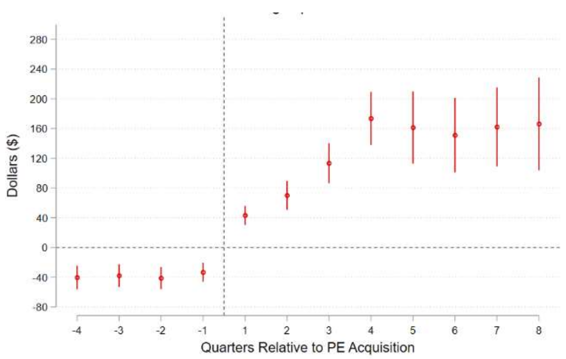

(d) Total number of E&M visits

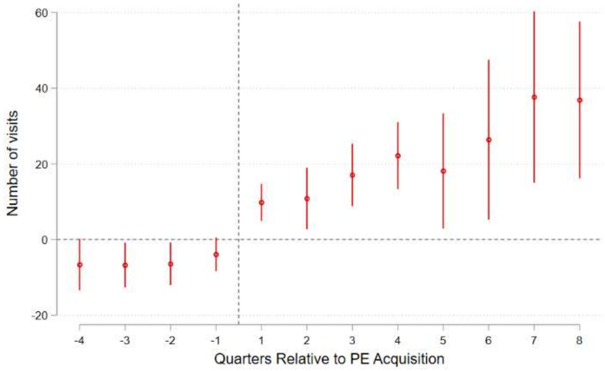

(e) Total number of encounters

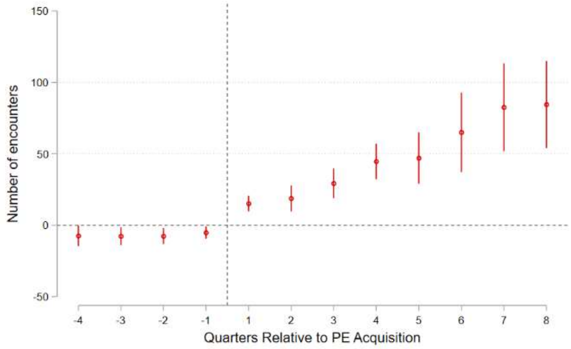

(f) Median HCC score

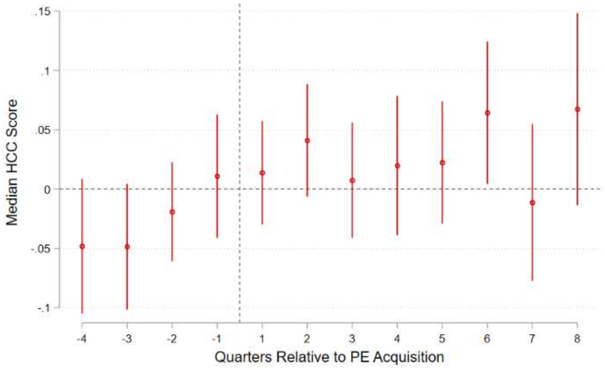

(g) E&M visits longer than 30 min (New)

(h) E&M visits longer than 30 min (Est)

© 2022 Singh et al. *JAMA Health Forum.*

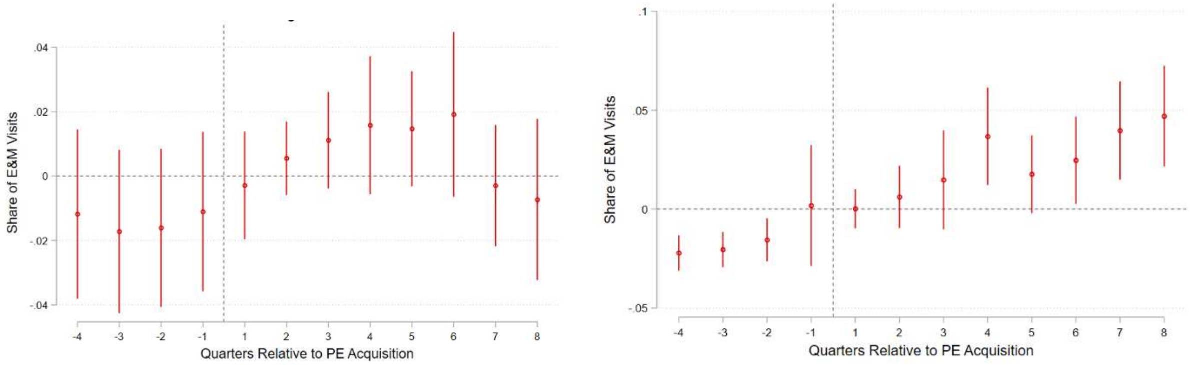

Notes: eFigure 3 presents event study coefficients corresponding to PE acquisition of physician practices. In the event study regression, we compared outcomes in PE-acquired practices with that of matched controls. Each point in the respective figure panels represents the coefficient obtained by estimating an event study regression which includes specialty and MSA fixed effects and with the unit of analysis at the practice-quarter level. The vertical dash line represents the quarter of acquisition that serves as the reference period. Event time 0 denoted the quarter of acquisition. We looked back up to 4 quarters prior to acquisition (event time -4, …, -1) and 8 quarters after (event time +1, +2, …, +8). Standard errors are clustered at the level of the matched cohort. Increases in confidence intervals in later quarters are explained by reductions in sample size.

© 2022 Singh et al. *JAMA Health Forum.*

eFigure 4: Changes in practice patterns associated with PE acquisition, by quarter (Practice and year fixed effects)

(a) Total spending per practice

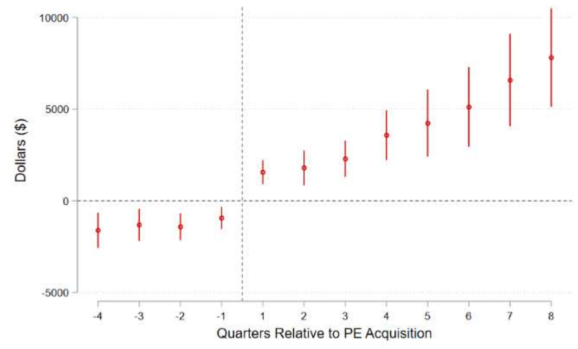

(b)Allowed amount per claim

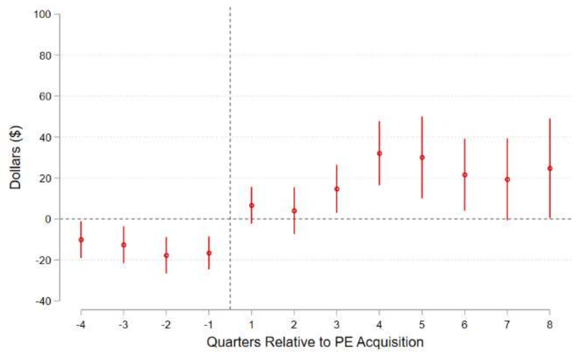

(c) Charges per claim

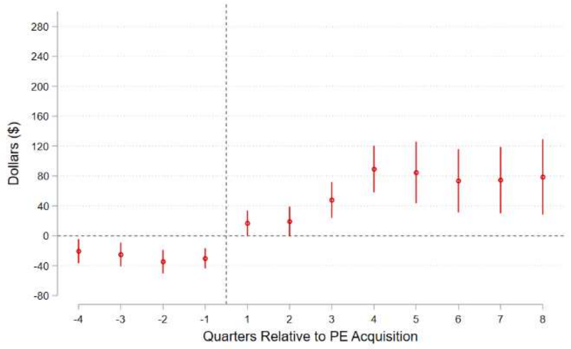

(d) Total number of E&M visits

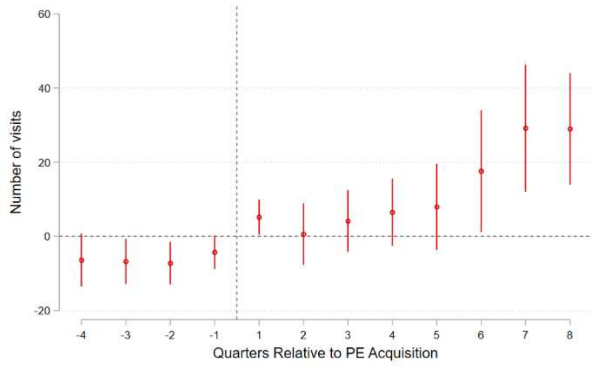

(e) Total number of encounters

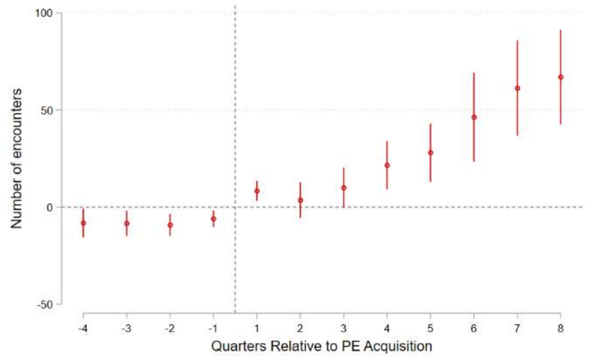

(f) Median HCC score

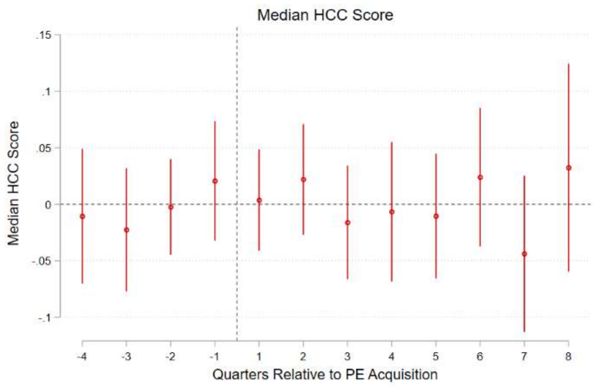

(g) E&M visits longer than 30 min (New)

(h) E&M visits longer than 30 min (Est)

© 2022 Singh et al. *JAMA Health Forum.*

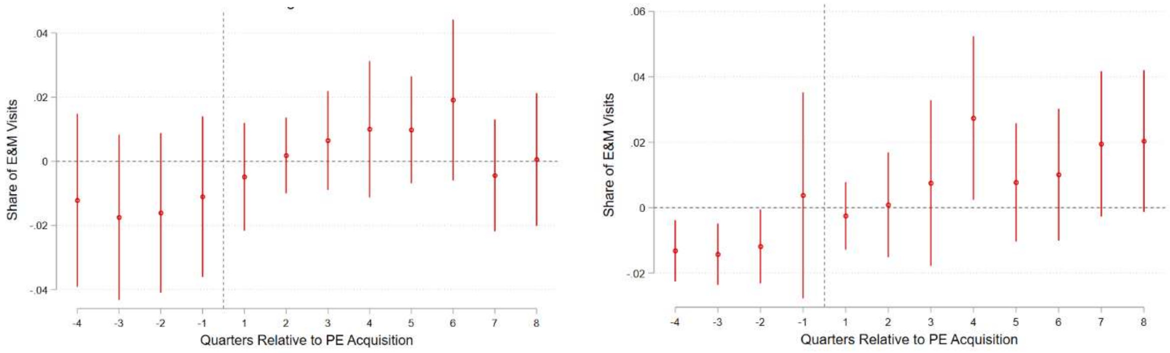

Notes: eFigure 4 presents event study coefficients corresponding to PE acquisition of physician practices. In the event study regression, we compared outcomes in PE-acquired practices with that of matched controls. Each point in the respective figure panels represents the coefficient obtained by estimating an event study regression which includes practice and year fixed effects and with the unit of analysis at the practice-quarter level. The vertical dash line represents the quarter of acquisition that serves as the reference period. Event time 0 denoted the quarter of acquisition. We looked back up to 4 quarters prior to acquisition (event time -4, …, -1) and 8 quarters after (event time +1, +2, …, +8). Standard errors are clustered at the level of the matched cohort. Increases in confidence intervals in later quarters are explained by reductions in sample size.

© 2022 Singh et al. *JAMA Health Forum.*

eFigure 5: Changes in practice patterns associated with PE acquisition, by quarter (Callaway & Sant'Anna (2021))

(a) Total spending per practice

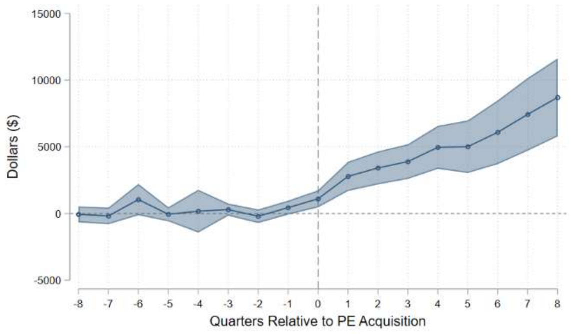

(b)Allowed amount per claim

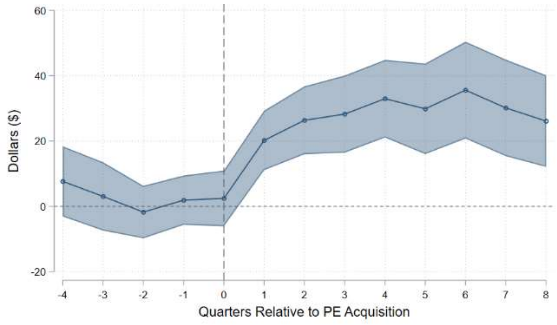

(c) Charges per claim

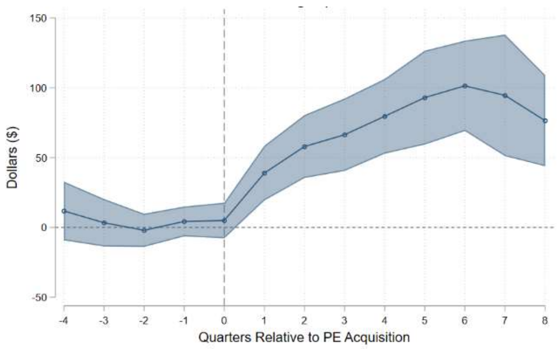

(d) Total number of E&M visits

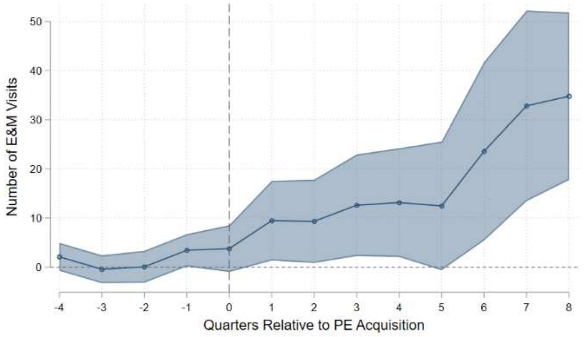

(e) Total number of encounters

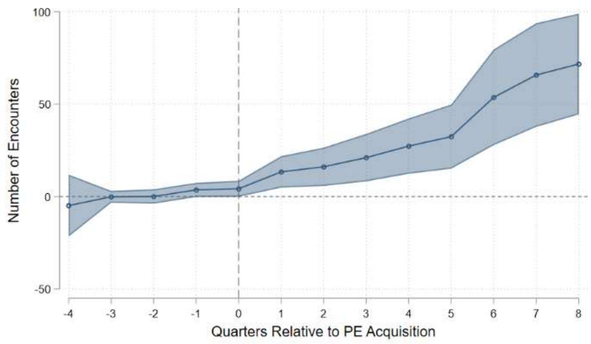

(f) Median HCC score

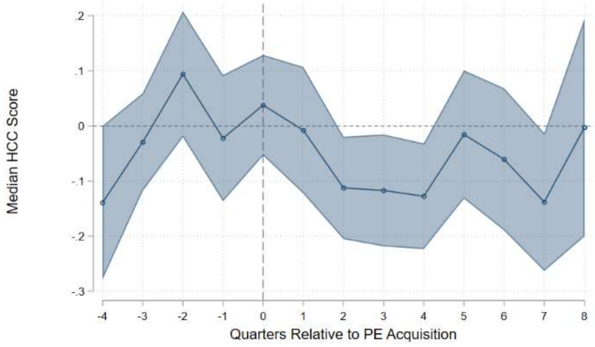

(g) E&M visits longer than 30 min (New)

(h) E&M visits longer than 30 min (Est)

© 2022 Singh et al. *JAMA Health Forum.*

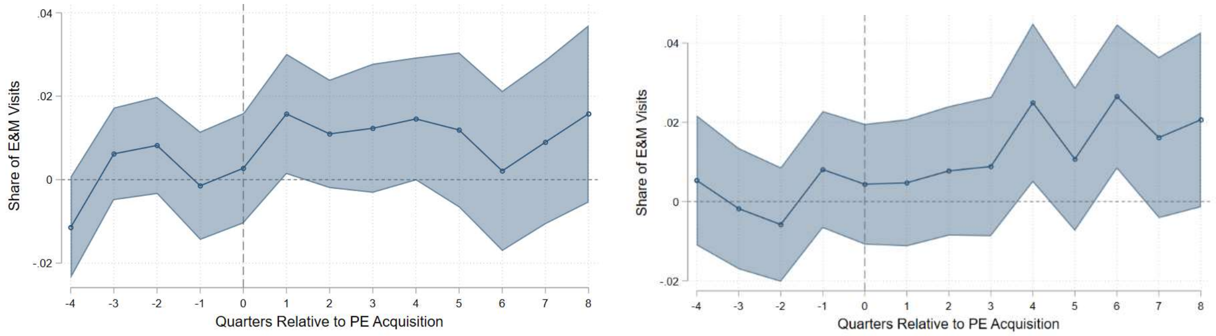

Notes: eFigure 5 presents event study coefficients corresponding to PE acquisition of physician practices. In the event study regression, we use the Callaway & Sant'Anna (2021) estimator that accounts for staggered adoption of treatment and heterogenous treatment effects. The vertical dash line represents the quarter of acquisition that serves as the reference period. Event time 0 denoted the quarter of acquisition. We looked back up to 4 quarters prior to acquisition (event time -4, …, -1) and 8 quarters after (event time +1, +2, …, +8). Standard errors are clustered at the level of the matched cohort. Increases in confidence intervals in later quarters are explained by reductions in sample size.

© 2022 Singh et al. *JAMA Health Forum.*

eFigure 6: Changes in practice patterns associated with PE acquisition, by quarter (Falsification Test)

(a) Total spending per practice

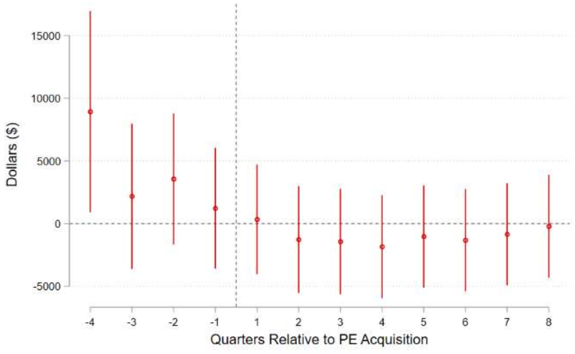

(b) Allowed amount per claim

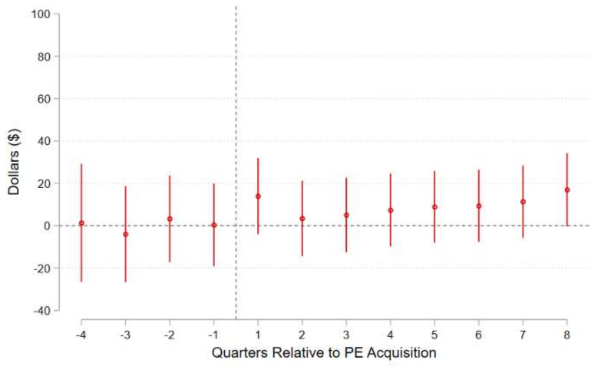

(c) Charges per claim

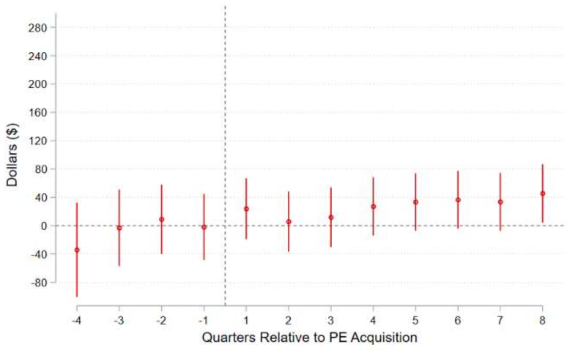

(d) Total number of E&M visits

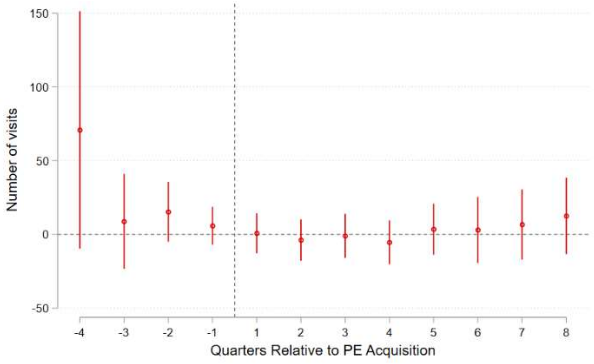

(e) Total number of encounters

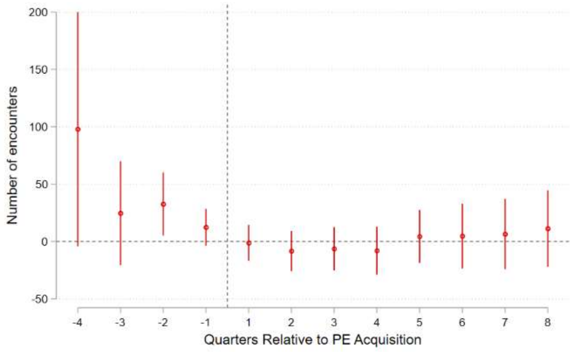

(f) Median HCC score

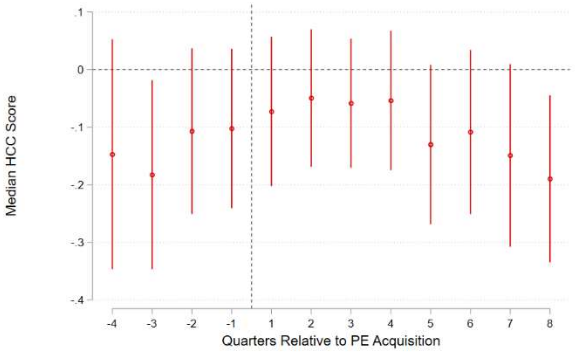

(g) E&M visits longer than 30 min (New)

(h) E&M visits longer than 30 min (Est)

© 2022 Singh et al. *JAMA Health Forum.*

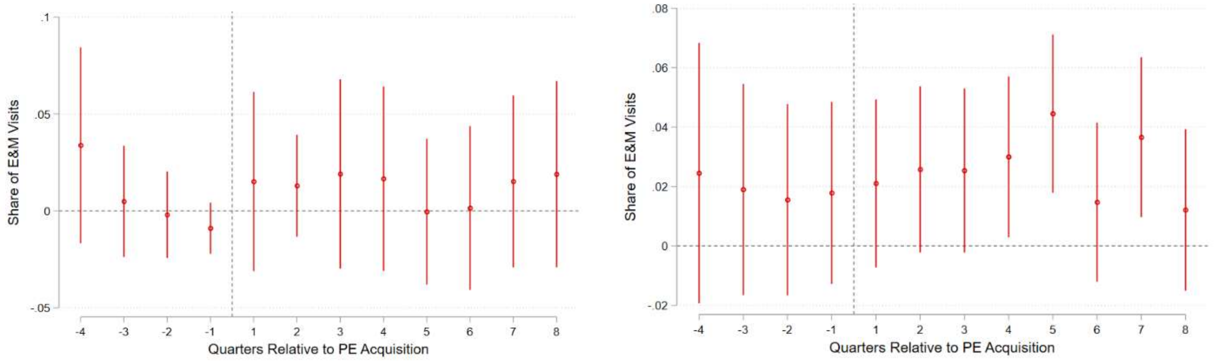

Notes: eFigure 6 presents event study coefficients corresponding to a falsification test where we assign a counterfactual acquisition date for all PE acquisition of physician practices and estimation treatment effects using a preacquisition (placebo) counterfactual acquisition date. The vertical dash line represents the quarter of acquisition that serves as the reference period. Event time 0 denoted the quarter of acquisition. We looked back up to 4 quarters prior to acquisition (event time -4, …, -1) and 8 quarters after (event time +1, +2, …, +8). Standard errors are clustered at the level of the matched cohort.

© 2022 Singh et al. *JAMA Health Forum.*

eTable 10: Differential change in practice patterns for PE and control practices (Dermatology only)

	PE		Controls		Unadjusted	Adjusted		
	Pre	Post	Pre	Post	Diff-in-diff	Diff-in-diff	% [95% C.I.]	p-value
Allowed amount per claim, $	120	149	166	202	-7	0.02	0.02 [-4.9, 4.9]	0.993
**Utilization**								
Unique patients	148	180	124	132	24	24.858	16.8 [3.3, 30.3]	0.015
New patients	79	106	62	67	22	21.933	27.7 [11.6, 43.8]	< 0.01
**Practice Patterns**								
Charge per claim, $	202	300	312	380	30	37.76	20.0 [9.6, 30.4]	< 0.01
Patient HCC score	0.87	0.88	1.03	1.08	-0.04	0.00881	4.5 [-2.7, 11.5]	0.779
E&M visits >30 minutes (established patients), %	0.15	0.19	0.15	0.16	0.02	0.0319	21.1 [7.2, 34.8]	< 0.01
E&M visits >30 minutes (new patients), %	0.24	0.24	0.26	0.27	0.01	0.24648	3.4 [-2.8, 9.5]	0.28
Evaluation & Management visits	189.82	226.03	162.33	170.90	16.25	43.15	7.0 [-13.5, 10.2]	0.503
Encounters	156.87	186.41	127.78	135.93	33.37	62.388	8.6 [-10.8, 27.9]	0.384

Notes: eTable 10 shows unadjusted and adjusted differential changes in outcome variables averaged at the practice level for dermatology practices acquired by PE. Post-acquisition period remains 8 quarters. E&M=Evaluation and Management, HCC=Hierarchical Condition Category. Diff-in-diff is the difference in differences between PE and controls or the differential change.

© 2022 Singh et al. *JAMA Health Forum.*

eTable 11: Differential change in practice patterns for PE and control practices (Gastroenterology only)

	PE		Controls		Unadjusted	Adjusted		
	Pre	Post	Pre	Post	Diff-in-diff	Diff-in-diff	% [95% C.I.]	p-value
Allowed amount per claim, $	328	512	294	398	80	114.09	32.2 [21.9, 42.5]	< 0.01
**Utilization**								
Unique patients	81	128	77	98	26	26.785	32.9 [16.6, 49.3]	< 0.01
New patients	54	88	47	60	21	32.931702	37.9 [21.1, 54.7]	< 0.01
**Practice Patterns**								
Charge per claim, $	578	901	553	731	145	224.9	35.7 [22.4,49.1]	< 0.01
Patient HCC score	1.87	1.98	1.98	2.23	-0.14	0.0046	0.3 [-7.6, 8.0]	0.957
E&M visits >30 minutes (established patients), %	0.30	0.31	0.31	0.30	0.02	0.02072	6.8 [0.9, 12.7]	0.023
E&M visits >30 minutes (new patients), %	0.27	0.30	0.25	0.27	0.01	0.0089	3.3 [-2.7, 9.3]	0.277
Evaluation & Management visits	113.21	179.77	112.53	143.31	16.25	15.117	10.9 [-16.8, 38.6]	0.439
Encounters	59.70	97.40	68.07	88.76	33.37	62.1	23.1 [-8.8, 55.1]	0.155

Notes: eTable 11 shows unadjusted and adjusted differential changes in outcome variables averaged at the practice level for gastroenterology practices acquired by PE. Post-acquisition period remains 8 quarters. E&M=Evaluation and Management, HCC=Hierarchical Condition Category. Diff-in-diff is the difference in differences between PE and controls or the differential change.

© 2022 Singh et al. *JAMA Health Forum.*

eTable 12: Differential change in practice patterns for PE and control practices (Ophthalmology only)

	PE		Controls		Unadjusted	Adjusted		
	**Pre**	**Post**	**Pre**	**Post**	**Diff-in-diff**	**Diff-in-diff**	**% [95% C.I.]**	**p-value**
Allowed amount per claim, $	209	284	162	221	16	11.87	7.1<br>[0.6, 13.5]	0.033
**Utilization**								
Unique patients	72	116	68	83	29	28.544	39.5<br>[17.9, 61.2]	< 0.01
New patients	31	65	28	40	22	20.975	67<br>[30.2, 103.8]	< 0.01
**Practice Patterns**								
Charge per claim, $	349	486	285	382	40	39.54	12.4<br>[2.9, 22.0]	0.011
Patient HCC score	1.16	1.17	1.21	1.28	-0.06	0.0527	4.5<br>[-2.7, 11.5]	0.219
E&M visits >30 minutes (established patients), %	0.18	0.18	0.17	0.18	0.02	-0.0183	-11.9<br>[-35.4, 11.5]	0.316
E&M visits >30 minutes (new patients), %	0.25	0.30	0.25	0.27	0.01	0.04353	21.0<br>[-23.7, 65.8]	0.355
Evaluation & Management visits	94.51	150.66	86.15	104.77	16.25	13.241	17.5<br>[-5.0, 39.9]	0.126
Encounters	20.46	30.01	22.19	26.97	33.37	190.65	44.0<br>[14.5, 73.5]	< 0.01

Notes: eTable 12 shows unadjusted and adjusted differential changes in outcome variables averaged at the practice level for ophthalmology practices acquired by PE. Post-acquisition period remains 8 quarters. E&M=Evaluation and Management, HCC=Hierarchical Condition Category. Diff-in-diff is the difference in differences between PE and controls or the differential change.

© 2022 Singh et al. *JAMA Health Forum.*

eFigure 7. Differential change in volume and spending of select services – Dermatology

(a) No. of services- Biopsies

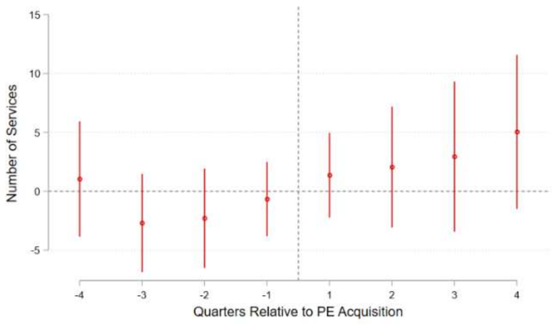

(b) Total Spending - Biopsies

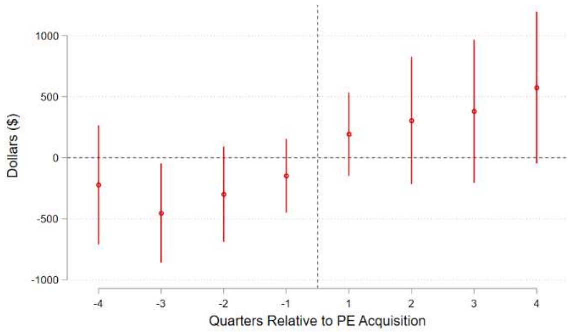

(c) No. of services- Pathology

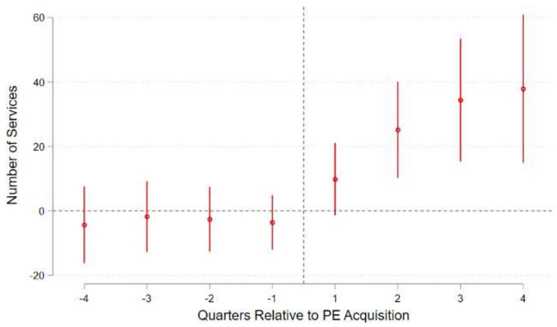

(d) Total Spending - Pathology

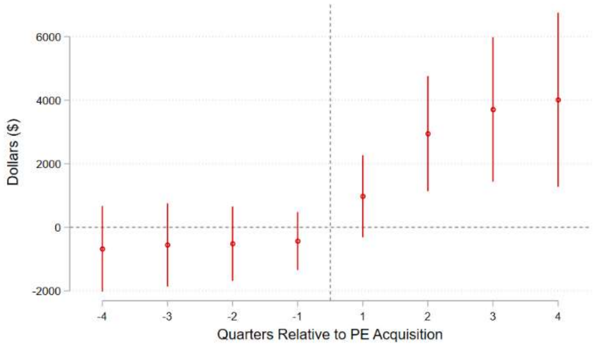

(e) No. of services- E&M Visits

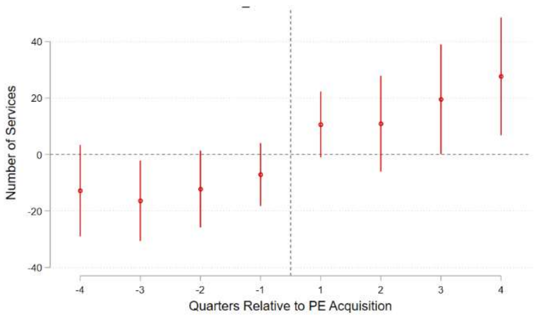

(f) Total Spending – E&M Visits

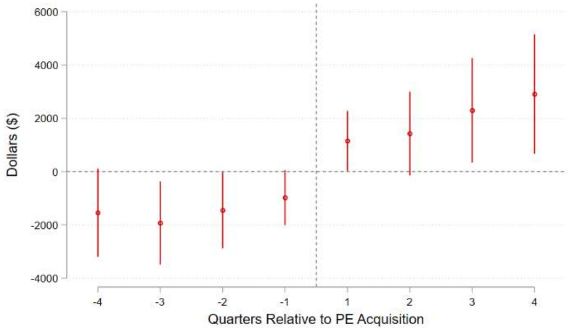

Notes: eFigure 7 presents event study coefficients corresponding to PE acquisition of dermatology physician practices. In the event study regression, we compared spending and volume for select services in PE-acquired practices with that of matched controls. Each point in the respective figure panels represents the coefficient obtained by estimating an event study regression which includes MSA fixed effects and with the unit of analysis at the practice-quarter level. Standard errors are clustered at the level of the matched cohort.

© 2022 Singh et al. *JAMA Health Forum.*

eFigure 8. Differential change in volume and spending of select services – Gastroenterology

(a) No. of services- Removal of tumors

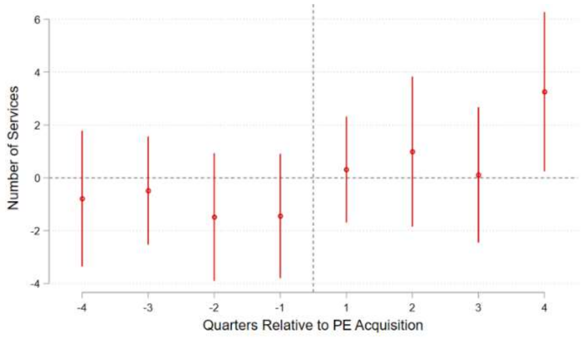

(b) Total Spending - Removal of tumors

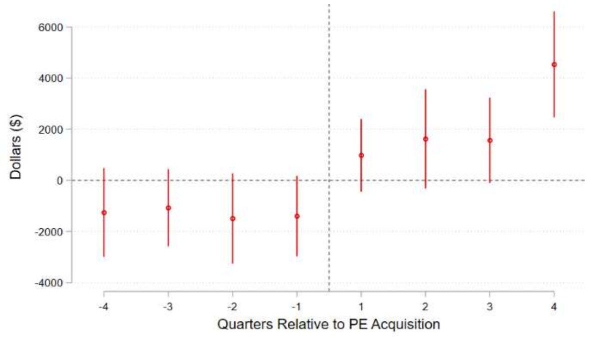

(c) No. of services- EGD

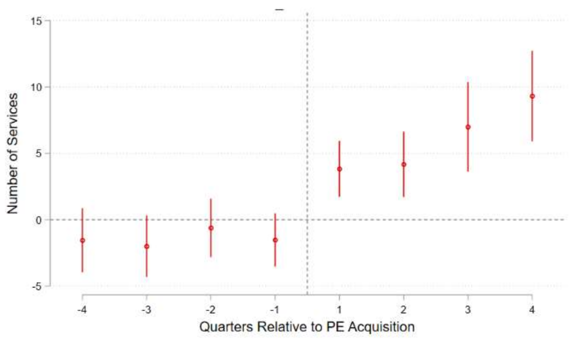

(d) Total Spending - EGD

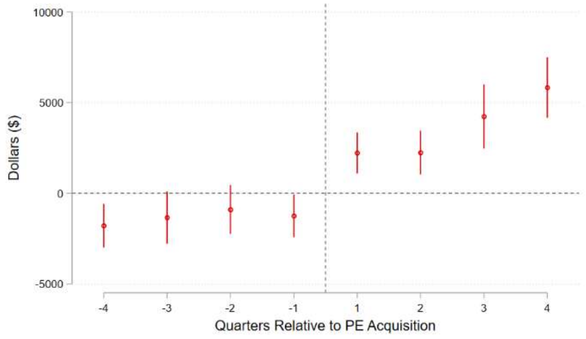

(e) No. of services- E&M Visits

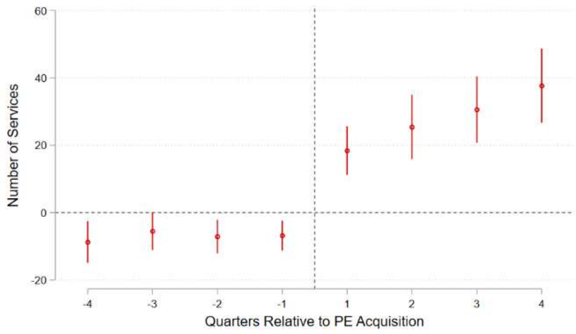

(f) Total Spending – E&M Visits

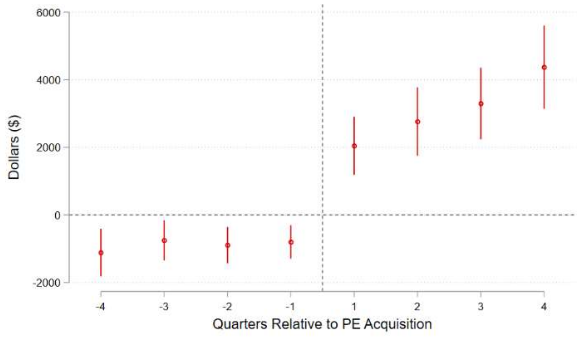

Notes: eFigure 8 presents event study coefficients corresponding to PE acquisition of gastroenterology physician practices. In the event study regression, we compared spending and volume for select services in PE-acquired practices with that of matched controls. Each point in the respective figure panels represents the coefficient obtained by estimating an event study regression which includes MSA fixed effects and with the unit of analysis at the practice-quarter level. Standard errors are clustered at the level of the matched cohort.

© 2022 Singh et al. *JAMA Health Forum.*

eFigure 9. Differential change in volume and spending of select services – Ophthalmology

(a) No. of services – Cataract Extraction

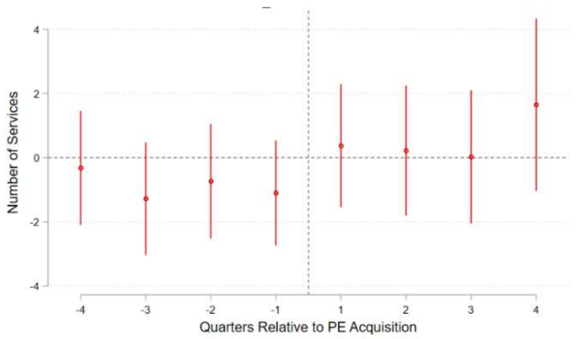

(b) Total Spending - Cataract Extraction

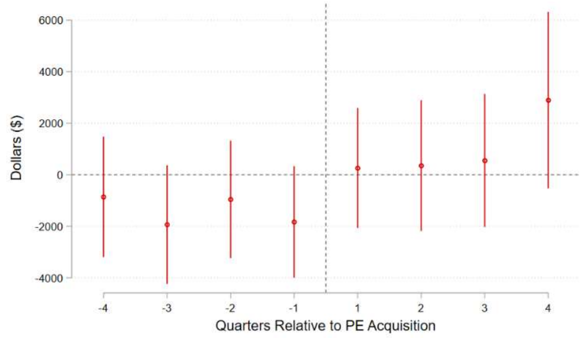

(c) No. of services- Diagnostic Imaging

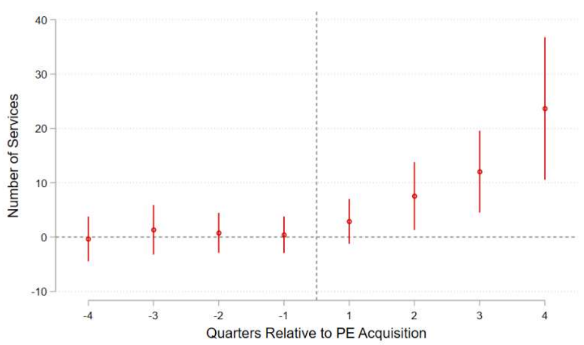

(d) Total Spending – Diagnostic Imaging

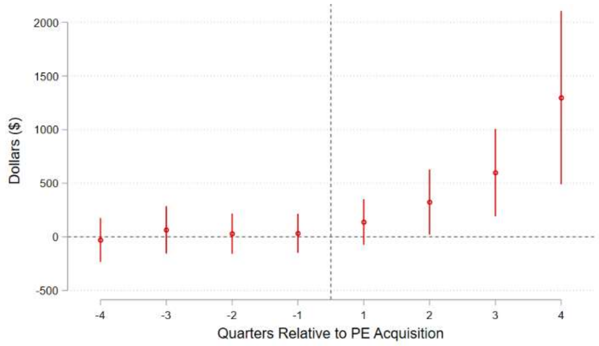

(e) No. of services- Eye Exams

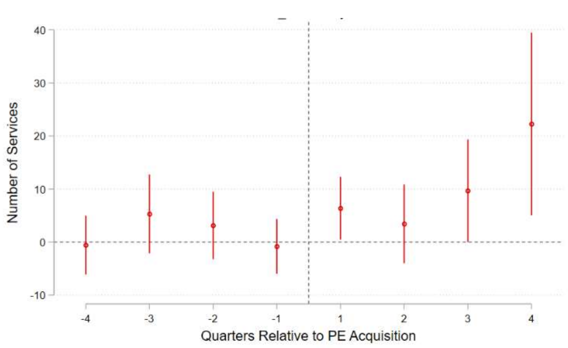

(f) Total Spending – Eye Exams

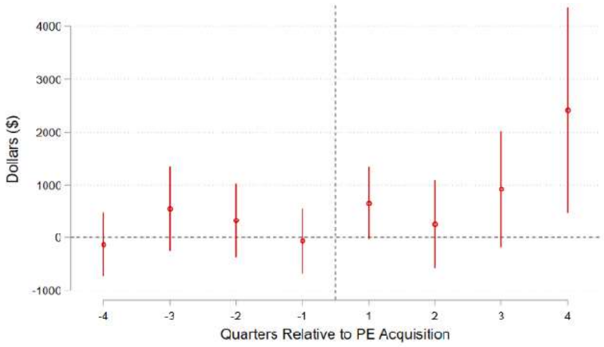

Notes: eFigure 9 presents event study coefficients corresponding to PE acquisition of ophthalmology physician practices. In the event study regression, we compared spending and volume for select services in PE-acquired practices with that of matched controls. Each point in the respective figure panels represents the coefficient obtained by estimating an event study regression which includes MSA fixed effects and with the unit of analysis at the practice-quarter level. Standard errors are clustered at the level of the matched cohort.

© 2022 Singh et al. *JAMA Health Forum.*
